# Supplementary material for: Exposed CendR Domain in Homing Peptide Yields Skin-Targeted Therapeutic in Epidermolysis Bullosa
Source: Mol Ther. 2020 May 20;28(8):1833–45. doi: 10.1016/j.ymthe.2020.05.017 (PMC7403337; doi:10.1016/j.ymthe.2020.05.017)
Supplement: Document S1. Figures S1–S6, Tables S1 and S2, and Supplemental Materials and Methods [file mmc1.pdf]

YMTHE, Volume *28*

Supplemental Information

Exposed CendR Domain in Homing Peptide Yields Skin-Targeted Therapeutic in Epidermolysis Bullosa

Toini Pemmari, Larisa Ivanova, Ulrike May, Prakash Lingasamy, Allan Tobi, Anja Pasternack, Stuart Prince, Olli Ritvos, Shreya Makkapati, Tambet Teesalu, Mitchell S. Cairo, Tero A.H. Järvinen, and Yanling Liao

Supplemental Figures

Figure S1

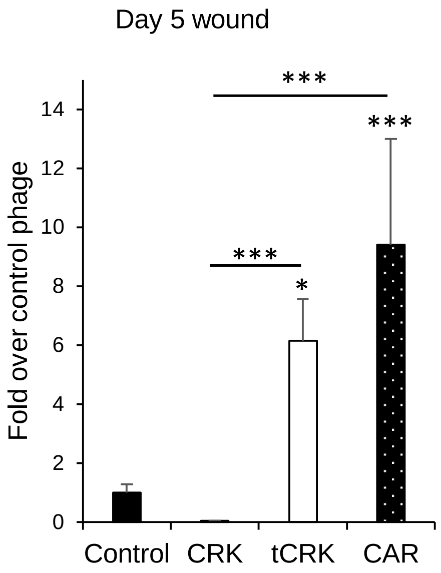

Figure S1: Homing of tCRK phage in 5 days old wound. The amounts are represented as fold over control phage. Error bars represent SEM, *p < 0.05, **p < 0.01, ***p < 0.001, Kruskal-Wallis test with Bonferroni post hoc, n = 14 for tCRK and CAR, n = 5 for CRK, and n = 11 for control phage.

Figure S2

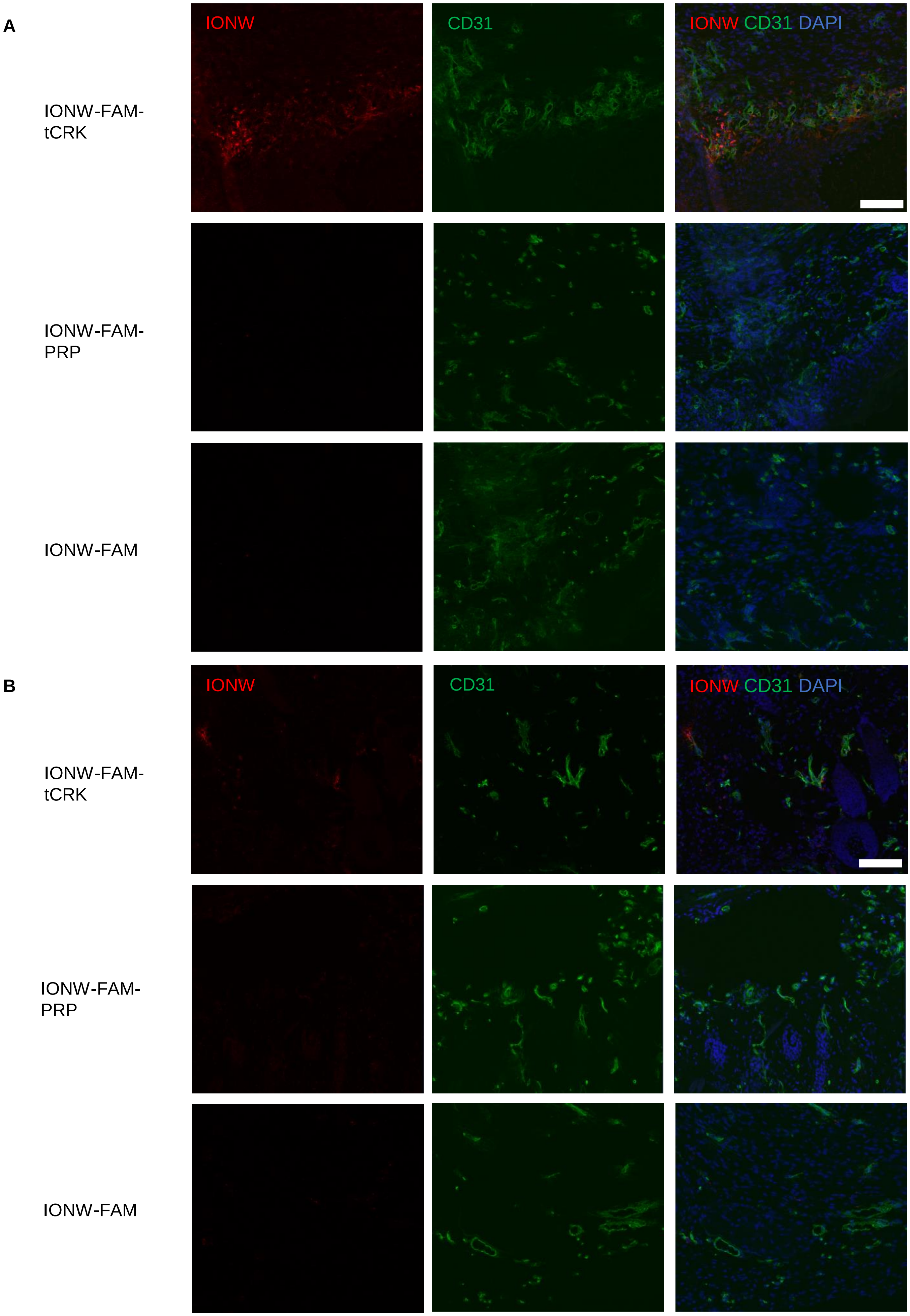
A
IONW-FAM-tCRK
IONW-FAM-PRP
IONW-FAM
IONW
CD31
IONW CD31 DAPI
B
IONW-FAM-tCRK
IONW-FAM-PRP
IONW-FAM
IONW
CD31
IONW CD31 DAPI

Figure S2: Homing of tCRK coated nanoparticles to skin wounds. Representative images of immunohistochemical staining of excision wound (A) and excision wound with splints (B) samples from Balb/c mice with anti-FITC (red) to detect the *i.v.* injected FAM-labeled IONWs coated with tCRK (12 mg/kg), a control peptide PRP (16 mg/kg) or no peptide (FAM alone, 13 mg/kg). The localization of blood vessels is depicted by anti-CD31 (green) and nuclei are stained with DAPI (blue). Scale bar 100 μm. Representative images from three independent experiments.

Figure S3

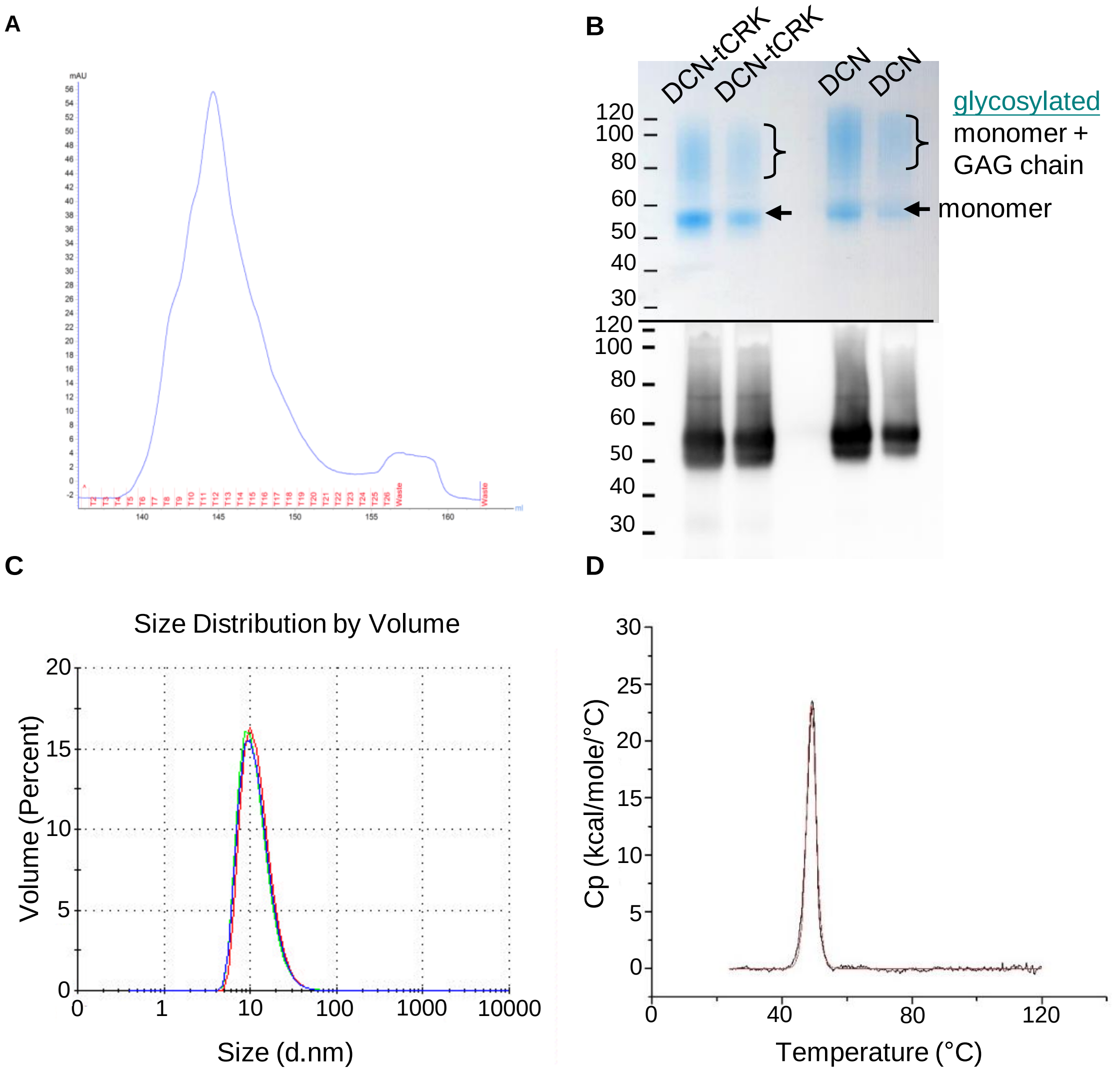

Figure S3: Recombinant protein production and characterization of DCN-tCRK. (A) An example of a purification chromatogram after the HisTrap HP column step on the Äkta Start shows one big peak, of which all peak fractions were used for further processing. (B) Coomassie-stained reduced SDS-Page gel (upper panel) and Western blot (lower panel) of purified DCN-tCRK are shown alongside the already published/characterized DCN. On the SDS gel 2 and 1 µg of protein were loaded; for Western blot analysis 1 and 0.5 µg of protein were applied. Monomeric forms of the proteins, as well as forms including the GAG side chains are visible. (C) Dynamic light scattering (DLS) measurements (n = 3) on the hydrodynamic diameter of DCN-tCRK. (D) Differential scanning calorimetry (DSC) curve for the melting temperature of DCN-tCRK.

Figure S4

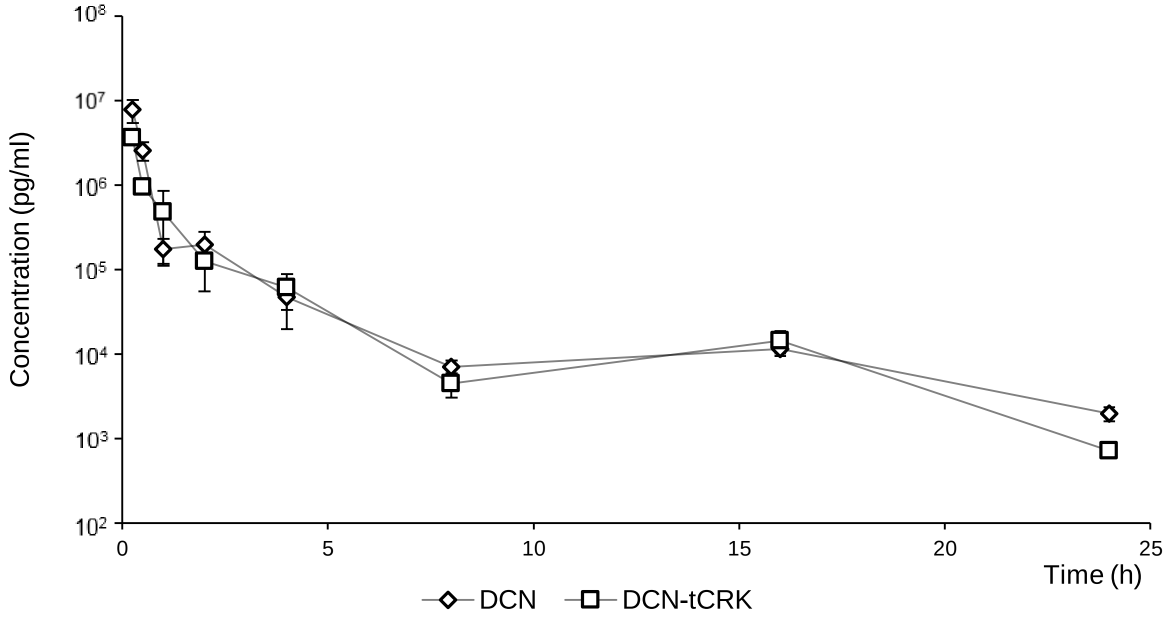

Figure S4: The pharmacokinetics of DCN-tCRK and DCN. 5 mg/kg of DCN-tCRK or DCN was injected *i.v.* Blood samples were gathered and analyzed with standard ELISA for human DCN from eight time points. Error bars represent SD, n = 4 per group.

Figure S5

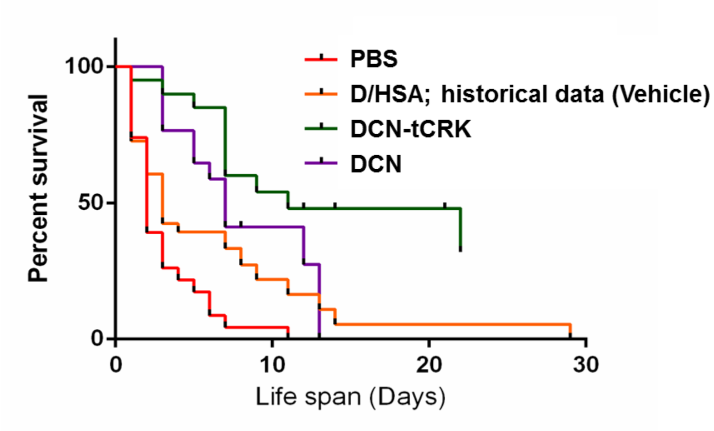

Figure S5: Kaplan-Meier survival analysis of the *col7a1*$^{-/-}$ mice comparing the historical survival after dextran/human serum albumin (D/HSA; median life span: 3 days; n = 29, orange line; historical data[1]) administration with the survival after DCN-tCRK (median life span: 11 days; n = 21, green line) and DCN (median life span: 7 days; n = 17, purple line) and PBS (median life span: 2 days; n = 24, red line) administration.

Figure S6

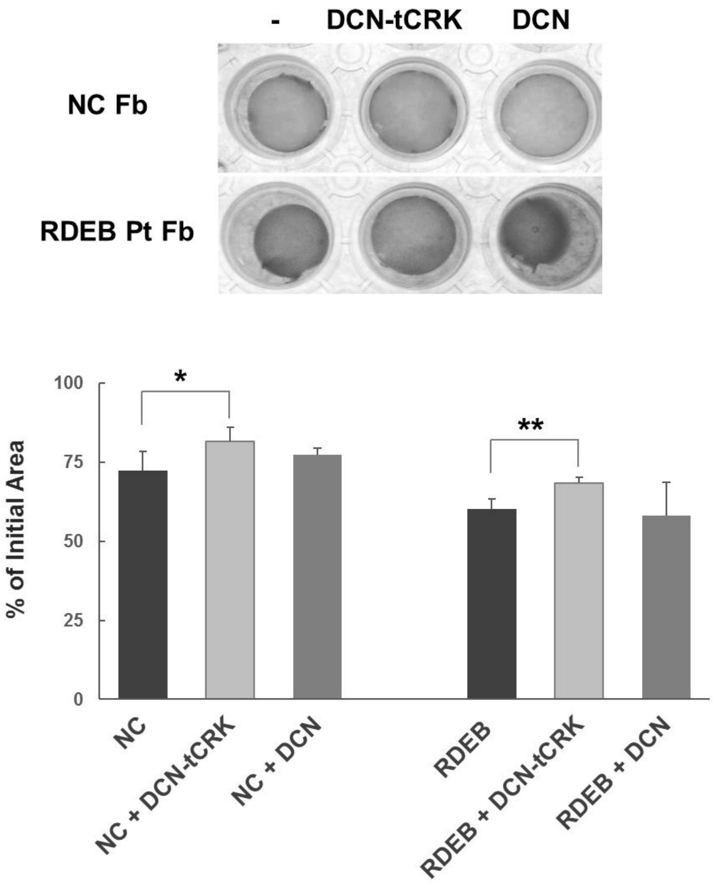

Figure S6: Collagen lattice contraction assay. Upper, representative images of human normal fibroblasts and RDEB patient-derived fibroblasts 48 hours after seeding in collagen gels, with and without addition of DCN and DCN-tCRK at a final concentration of 75 µM. Bottom, contraction of collagen gels, calculated as percentage of contraction compared with the initial area. Data (n = 3) are presented as mean ± SEM. * $p < 0.05$, ** $p < 0.001$.

Supplemental Tables

Table S1: The sequence of human DCN and the tCRK sequence in the C-terminus analyzed by mass spectrometry. The green letters indicate the peptides that were found to be specific to human DCN and the blue letters indicate the specific C-end terminus including the tCRK sequence, which is further indicated with bold.

N	Unused	Total	% Cov	Accession #	Name	Peptides (95%)
1	54,45	54,45	45,7	sp|P07585|PGS2 _HUMAN	Decorin OS=Homo sapiens GN=DCN PE=1 SV=1	48
					MKATIILLLLAQVSWAGPFQQRGLFDFMLEDEAS<br>GIGPEVPDDRDFEPSLGPVCPFRCQCHLRVVQCSD<br>LGLDKVPKDLPPDTTLLDLQNNKITEIKDGDFKNL<br>KNLHALILVNNKISKVSPGAFTPLVKLERLYLSKN<br>QLKELPEKMPKTLQELRAHENEITKVRKVTFNGL<br>NQMIVIELGTNPLKSSGIENGAFQGMKKLSYIRIA<br>DTNITSIPQGLPPSLTELHLDGNKISRVDAASLKGL<br>NNLAKLGLSFNSISAVDNGSLANTPHLRELHLDN<br>NKLTRVPGGLAEHKYIQVVYLHNNNISVVGSSDF<br>CPPGHNTKKASYSGVSLFSNPVQYWEIQPSTFRCV<br>YVRSAIQLGNYK	
1	66	66	46,7	sp|P07585|PGS2 _HUMAN	Decorin OS=Homo sapiens OX=9606 GN=DCN PE=1 SV=1	63
					MKATIILLLLAQVSWAGPFQQRGLFDFMLEDEAS<br>GIGPEVPDDRDFEPSLGPVCPFRCQCHLRVVQCSD<br>LGLDKVPKDLPPDTTLLDLQNNKITEIKDGDFKNL<br>KNLHALILVNNKISKVSPGAFTPLVKLERLYLSKN<br>QLKELPEKMPKTLQELRAHENEITKVRKVTFNGL<br>NQMIVIELGTNPLKSSGIENGAFQGMKKLSYIRIA<br>DTNITSIPQGLPPSLTELHLDGNKISRVDAASLKGL<br>NNLAKLGLSFNSISAVDNGSLANTPHLRELHLDN<br>NKLTRVPGGLAEHKYIQVVYLHNNNISVVGSSDF<br>CPPGHNTKKASYSGVSLFSNPVQYWEIQPSTFRCV<br>YVRSAIQLGNYKGSEF**CRKDK**	
					GREEN: Peptides that were found to be specific to human DCN; BLUE: specific for the C-terminus including the tCRK sequence (CRKDK)	

Table S2: Fold changes of gene expression in vehicle, DCN and DCN-tCRK treated *col7a*$^{-/-}$ skin relative to the WT and P values. N/A indicates average threshold cycle either not determined or greater than the defined cut-off. The genes that are significantly upregulated as compared to the WT are bolded and the genes that are significantly upregulated only in DCN-treated *col7a*$^{-/-}$ skin were marked as red.

**Gene Symbols**	**Vehicle RDEB vs WT**		**DCN-tCRK RDEB vs WT**		**DCN RDEB vs. WT**	
	**Fold changes**	**P value**	**Fold changes**	**P value**	**Fold changes**	**P value**
Acta2	1.55	0.695	0.84	0.672	N/A	N/A
Actc1	1.19	0.633	1.36	0.815	0.65	0.426
Angpt1	1.54	0.545	0.72	0.927	2.94	0.271
Ccl12	8.28	0.375	N/A	N/A	N/A	N/A
Ccl7	0.66	0.701	0.74	0.594	**3.07**	**0.001**
Cd40lg	0.85	0.783	N/A	N/A	N/A	N/A
Cdh1	2.58	0.076	1.76	0.439	3.20	0.062
Col14a1	0.89	0.690	0.84	0.737	1.02	0.751
Col1a1	1.79	0.405	1.45	0.396	1.16	0.893
Col1a2	0.82	0.897	0.53	0.830	1.2	0.722
Col3a1	1.06	0.885	0.6	0.74	1.71	0.501
Col4a1	0.94	0.487	0.25	0.356	N/A	N/A
Col4a3	2.96	0.203	1.08	0.655	3.16	0.068
Col5a1	4.47	0.195	1.21	0.582	1.12	0.226
Col5a2	1.24	0.554	0.61	0.795	1.34	0.572
Col5a3	1.86	0.370	1.8	0.410	1.83	0.228
Csf2	0.91	0.849	0.7	0.762	**45.86**	**0.004**
Csf3	1.36	0.439	2.19	0.332	**7.04**	**0.049**
**Ctgf**	**3.26**	**0.037**	2.44	0.281	**4.55**	**0.050**
**Ctnnb1**	**3.08**	**0.009**	2.25	0.349	**3.79**	**0.009**
**Ctsg**	**3.15**	**0.002**	1.77	0.292	**9.23**	**0.021**
Ctsk	1.7	0.266	0.69	0.897	1.4	0.422
Ctsl	2.25	0.285	1.01	0.883	2.42	0.207
Cxcl1	4.97	0.106	2.05	0.326	12.49	0.085
Cxcl11	N/A	N/A	N/A	N/A	N/A	N/A
**Cxcl3**	**3.41**	**0.009**	1.98	0.073	**26.44**	**0.047**
Cxcl5	1.25	0.449	1.35	0.482	**7.03**	**0.023**
Egf	0.66	0.218	0.54	0.816	0.67	0.031
**Egfr**	**4.68**	**0.015**	1.75	0.367	**2.20**	**0.039**
F13a1	1.96	0.493	0.82	0.747	1.78	0.484
F3	3.04	0.114	1.74	0.423	2.99	0.172
Fga	N/A	N/A	N/A	N/A	N/A	N/A
Fgf10	1.84	0.164	1.15	0.543	1.56	0.317
Fgf2	2.2	0.125	1.17	0.633	3.49	0.164
Fgf7	1.23	0.761	1.1	0.730	1.13	0.951
Hbegf	2.69	0.154	1.11	0.658	**4.01**	**0.026**
Hgf	3.36	0.377	0.25	0.259	8.12	0.374
Ifng	N/A	N/A	N/A	N/A	N/A	N/A
Igf1	1.22	0.606	0.71	0.694	1.79	0.337
Il10	2.00	0.408	1.1	0.979	3.26	0.235
Il1b	4.12	0.262	1.1	0.608	100.49	0.132
Il2	N/A	N/A	N/A	N/A	N/A	N/A
**Il4**	**4.29**	**0.015**	2.08	0.302	**13.75**	**0.006**
Il6	1.62	0.358	1.34	0.571	8.75	0.082
Il6st	1.81	0.313	1.58	0.481	2.35	0.141
Itga1	1.58	0.42	0.95	0.909	1.2	0.913
Itga2	2.24	0.16	1.45	0.547	2.17	0.197
Itga3	4.57	0.104	2.17	0.172	**2.01**	**0.003**
Itga4	2.26	0.746	0.447	0.304	N/A	N/A

Itga5	3.37	0.207	1.62	0.397	2.39	0.127
Itga6	2.41	0.106	1.39	0.507	4.31	0.295
Itgav	2.25	0.148	1.32	0.557	2.14	0.159
Itgb1	2.29	0.178	0.69	0.769	**2.90**	**0.048**
**Itgb3**	**1.57**	**0.001**	0.57	0.084	1.24	0.391
**Itgb5**	**5.78**	**0.050**	3.79	0.266	3.29	0.181
Itgb6	1.26	0.228	0.62	0.680	2.68	0.329
Mapk1	2.01	0.167	1.42	0.488	**3.44**	**0.012**
**Mapk3**	**2.18**	**0.040**	1.33	0.504	1.75	0.267
Mif	0.4	0.709	1.64	0.439	1.1	0.986
Mmp1a	1.27	0.451	0.95	0.713	0.26	0.013
Mmp2	N/A	N/A	N/A	N/A	N/A	N/A
Mmp7	N/A	N/A	N/A	N/A	N/A	N/A
Mmp9	1.18	0.879	0.71	0.810	2.33	0.355
Pdgfa	1.27	0.785	1.17	0.587	4.35	0.065
Plat	1.31	0.603	1.41	0.416	0.93	0.814
**Plaur**	**3.59**	**0.002**	2.72	0.342	**20.26**	**0.041**
Plau	2.41	0.127	1.39	0.464	3.94	0.122
Plg	N/A	N/A	N/A	N/A	N/A	N/A
Pten	3.79	0.203	0.605	0.159	4.59	4.59
Ptgs2	1.84	0.79	0.435	0.318	8.44	0.167
Rac1	1.42	0.860	0.94	0.693	0.65	0.752
Rhoa	3.54	0.101	2.97	0.376	**7.69**	**0.043**
Serpine1	5.63	0.111	2.42	0.309	5.00	0.062
Stat3	3.8	0.065	2.19	0.377	4.65	0.172
Tagln	1.32	0.156	0.56	0.801	0.78	0.908
Tgfa	N/A	N/A	N/A	N/A	N/A	N/A
**Tgfb1**	**2.38**	**0.040**	1.6	0.332	2.33	0.361
**Tgfbr3**	**8.11**	**0.006**	3.93	0.222	**6.46**	**0.041**
Timp1	0.29	0.194	0.22	0.168	2.25	0.331
**Tnf**	**3.92**	**0.024**	1.1	0.683	**12.16**	**0.049**
Vegfa	6.54	0.129	3.02	0.369	3.4	0.274
Vtn	1.22	0.721	0.75	0.985	1.23	0.780
Wisp1	1.26	0.693	0.70	0.890	1.23	0.781
Wnt5a	1.89	0.212	1.32	0.493	1.08	0.895

Supplemental Methods

Generation of phage clones

The following oligonucleotide primers (TAG Copenhagen, Copenhagen, Denmark), expressing the indicated peptides, were used to prepare phage clones: tCRK (CRKDK):5´- AAT TCT TGC CGC AAA GAT AAA TAA GGA -3´and 5´- AGC TTC CTT ATT TAT CTT TGC GGC AAG-3´, CRK (CRKDKC): 5´- AAT TCC TGC CGG AAG GAT AAG TGC TA -3´and 5´- AGC TTA CGA CTT ATC CTT CCG GCA GG-3´, CAR (CARSKNKDC): 5´- AAT TCC TGC GCA CGT TCG AAG AAC AAA GAT TGC TA -3´and 5´- AGC TTA GCA ATC TTT GTT CTT CGA ACG TGC GCA GG-3´, where the 5´- end is phosphorylated. The random primer sequences for the negative control phage were 5´- AAT TCC TGC CTT TTG GGG AAG AAT TCG TGC TA -3´and 5´- AGC TTA GCA CGA ATT CTT CCC CAA AAG GCA GG-3´. The oligonucleotide mixture was heated to 95 °C, allowed to cool down to 20 °C in 75 minutes and then cloned into the T7Select 415-1b vector according to the manufacturer's instructions (Merck Millipore, Madison, WI). Individual phage plaques were isolated from the bacterial culture plate and the insert coding region of each recombinant phage was amplified by PCR and checked by DNA sequencing to confirm the clones used for the homing studies as previously described.[2]

Peptide synthesis

Peptides were synthesized with an automated peptide synthesizer by using standard solid-phase fluorenylmethoxycarbonyl chemistry. During synthesis, the peptides were labeled with fluorescein amitide (FAM) using an amino-hexanoic acid spacer as described previously.[3]

Preparation and characterization of iron oxide nanoworms (IONWs)

The iron oxide nanoworms (IONWs) were prepared based on a previously published protocol.[4] Briefly, 0.63 g of $FeCl_3•6H_2O$ (Sigma-Aldrich #44944) and 0.25 g of $FeCl_2•4H_2O$ (Sigma-Aldrich #44939) were mixed with 4.5 g of Dextran T20 (Pharmacosmos) in 30 ml of deionized water (Millipore). The reaction mixture was cooled to 0 °C. Under a steady flow of nitrogen and vigorous stirring, 1 ml of 28% aqueous ammonium hydroxide (Sigma-Aldrich #338818) was added over 45 minutes. Next, the reaction mixture was heated at 80 °C for 1 h, and cooled to room temperature (RT). After diluting with 90 ml of deionized water, the mixture was centrifuged in 50 ml Falcon tubes at 335 G for 20 min (RT) to remove larger aggregates. The colloidal suspension was transferred to 100,000 MWCO centrifugal filters (Millipore), and centrifuged at 760 G for 30 min (4 °C) – this washing step was repeated 4 times. The dextran was crosslinked with epichlorohydrin (Sigma-Aldrich #540080) in strongly basic conditions (addition of 5 M aqueous NaOH solution). After removing excess epicholohydrin and NaOH with 100,000 MWCO centrifugal filters, the IONWs were aminated with 28% aqueous ammonium hydroxide, dialysed for 48 h, and stored at 4 °C.

FAM coupled tCRK (CRKDK) or scrambled CendR peptide PRP (RPAAPRP) or FAM alone were coupled to the IONWs through a maleimide-PEG(5000)-NHS linker (Jenkem). IONWs were incubated with the linker for 1 h at RT with stirring, washed with PBS in aforementioned centrifugal filters, and then the peptide with free cysteine was added (in excess). After an overnight incubation at RT and final washes, the IONWs were filtered through a 0.22 μm filter, and used within the next week.

Nanoparticle (IONW) targeting study

The nanoparticles (IONWs) coated with either FAM-coupled tCRK, FAM-coupled PRP or FAM alone were dissolved in DPBS. Mice with either 8 or 9 days old excisional wounds or 10 or 11 days old excisional splint wounds were injected with IONW-tCRK (12 mg/kg), IONW-PRP (16 mg/kg) or IONW-FAM (13 mg/kg) intravenously through the tail vein. Five hours after the injection, the mice were perfused with phosphate buffered saline (PBS) containing 1 % bovine serum albumin (BSA) while under deep anesthesia (ketamine-medetomidine according to weight). Skin and skin wounds were excised, embedded in Tissue-Tek OCT medium (Sakura Europe, Alphen aan den Rijn, The Netherlands) and snap-frozen in liquid nitrogen. The samples were then cut and fixed with acetone for immunohistochemistry analysis (IHC). To determine the localization of the IONWs, the sections were double-immunostained with rabbit anti-fluorescein isothiocyanate (FITC) antibody (#71-1900, Invitrogen, Carlsbad, CA) followed by anti-rabbit secondary Alexa Fluor 594 (A11007, Invitrogen, Carlsbad, CA) and with rat anti-mouse CD31 antibody (BD550274, BD Biosciences, San Jose, CA) followed by anti-rat secondary Alexa Fluor 488 (A11070, Life Technologies, Carlsbad, CA). Images were acquired with Zeiss LSM 780 Laser Scanning Confocal Microscope using the same settings throughout the experiments.

Cloning of decorin fusion proteins

Human decorin cDNA[5] without the native signal and pro-peptide sequence were cloned into the mammalian expression vector pEFIRES-P.[6] The tCRK wound homing peptide cDNA was cloned to the C-terminus of decorin flanked by a stop-codon. A 6XHis-tag was cloned to the N-terminus ahead of decorin. The construct was assembled by using the PIPE method[7]. For transformation NEB 5-alpha competent *E. coli* (high efficiency) cells were used (C2987H; New England Biolabs Ipswich, MA) according to the manufacturer's instructions. For plasmid purification (Mini-Prep), PCR purification and agarose gel purification, kits from Qiagen (Hilden, Germany) were used. DCN naturally forms a dimer [8]. The protein sequence of a monomeric 6XHistag-DCN-tCRK fusion protein is: G H H H H H H D E A S G I G P E V P D D R D F E P S L G P V C P F R C Q C H L R V V Q C S D L G L D K V P K D L P P D T T L L D L Q N N K I T E I K D G D F K N L K N L H A L I L V N N K I S K V S P G A F T P L V K L E R L Y L S K N Q L K E L P E K Met P K T L Q E L R A H E N E I T K V R K V T F N G L N Q Met I V I E L G T N P L K S S G I E N G A F Q G Met K K L S Y I R I A D T N I T S I P Q G L P P S L T E L H L D G N K I S R V D A A S L K G L N N L A K L G L S F N S I S A V D N G S L A N T P H L R E L H L D N N K L T R V P G G L A E H K Y I Q V V Y L H N N N I S V V G S S D F C P P G H N T K K A S Y S G V S L F S N P V Q Y W E I Q P S T F R C V Y V R S A I Q L G N Y K G S E F C R K D K Stop.

A schematic map of the DCN-tCRK fusion protein is shown in Fig. 2.

Recombinant protein purification

Cell culture supernatants were filtered and degassed on ice through a 0.45 µm filter unit (Corning #430514, Corning, NY). The 6XHis-tagged proteins were purified by Ni-NTA- IMAC via a two-step purification protocol using first a HisTrap Excel column followed by a HisTrap HP column on the Äkta Start chromatography system (GE Healthcare, Munich, Germany) according to the manufacturer's instructions in a 4 °C cold cabinet. Buffers were prepared from the His Buffer Kit (GE Healthcare/VWR (11-0034-00). All buffers were filtered and degassed.

The HisTrap Excel column eluate was diluted in 20 mM sodium phosphate buffer (pH 7.4) with 0.5 M NaCl to a final imidazole concentration of 30 mM, and then further purified on a HisTrap HP column, with a 35 mM imidazole wash and a gradient elution up to 300 mM imidazole (Fig. S3 includes an example of such a purification chromatogram). The peak fractions were analyzed on a SDS NuPAGE 4-12 % gradient gel (Life Technologies/Thermo Fisher Scientific, Waltham, MA) and visualized via PageBlue Protein Staining Solution (Thermo Fisher Scientific, Waltham, MA).

Selected peak fractions were pooled and dialyzed against cold TBS buffer (pH 7.6) using 50 kDa MWCO Float-A-Lyzers (Fisher Scientific/Spectrum Labs), before concentration via 10 kDa MWCO VivaSpin 6 tubes (GE Healthcare). Samples were filter sterilized (Ultrafree-MC GV Centrifugal Filter 0.22 µm, Millipore, Burlington, MA) and the protein concentration measured at A280 nm via Nanodrop (Thermo Fisher Scientific, Waltham, MA). All steps were performed at 4 °C or on ice. Sterile Tween-20 was added to a final concentration of 0.05% to prevent aggregation, before freezing aliquots rapidly at -80 °C.

Recombinant protein was verified by SDS Page and Western blotting. BioRad's wet tank Mini-PROTEAN Trans-Blot Cell system was used (according to the manufacturer's instructions). A PVDF membrane was probed with a primary murine antibody against human decorin (MAB143, R&D Systems, Minneapolis, MN) according to the manufacturer's protocol. A secondary horseradish peroxidase-coupled anti-mouse antibody from Cell Signaling Technology was used. Chemiluminescent blot images were captured via ImageQuant LAS 4000 mini (GE Healthcare).

Biophysical protein analysis

The hydrodynamic diameter was measured by Dynamic Light Scattering (DLS) using a Zetasizer Nano ZS instrument (Malvern Instruments Ltd, Worchestershire, UK). The DCN-tCRK protein sample was diluted 1:5 in TBS buffer. Three 10X10 s measurements were performed at 25 °C. Data were analyzed using the Zetasizer software v7.11 (Malvern Instruments Ltd.) via the protein analysis model (non-negative least squares analysis followed by L-cuve) and size distribution by volume.

The unfolding temperature of DCN-tCRK was determined using the VP-Capillary DSC (differential scanning calorimetry) instrument (GE Healthcare, Microcal Inc./Malvern Instruments Ltd.) in TBS buffer (50 mM Tris-Cl, 150 mM NaCl, pH 7.5) with a protein concentration of 0.2 mg/ml. All solutions were degassed. Samples were heated from 20 °C to 130 °C at a scanning rate of 2 °C/min. Feedback mode was set to 'low' and the filter period was 5 s. The melting temperature Tm (transition midpoint) was calculated by a Non-2-state fitting model using Origin 7.0 DSC software suite (Microcal Inc.).

Expressed recombinant DCN-tCRK protein was identified from the monomeric gel band using Eksigent 425 NanoLC coupled with Sciex high speed TripleTOF™ 5600+ mass spectrometer. After isolation of gel band and Coomassie stain removal protein was then subjected to reduction (TCEP, 25 mM), alkylation (iodoacetamide, 0.5 M), and trypsin digestion as described in detail in Vähätupa et. al., 2018.[9] After trypsin digestion peptides were diluted to 14 µl of sample buffer (2 % acetonitrile, 0.1 % formic acid) and 1 µl of sample was injected to the triple TOF mass spectrometry.

In vitro binding analyses

In vitro binding of DCN-tCRK and peptides to NRP-1 was analyzed using ELISA analysis. 96-well, black FLUOTRAC™ 600, high binding plates (Greiner Bio-One, Kremsmünster, Austria) were coated with 100µL/well of 100 µg/ml DCN-tCRK in PBS at 4 °C overnight. 10 µg/well RPARPAR and RPAPRARA peptides were coated in parallel as a positive and negative control, respectively. BSA was used as an immobilization control. The plates were washed 3 times with phosphate buffered saline (PBS) and blocked for 1 h at 37 °C with 300 µl of blocking solution (1XPBS, 1% BSA, 0.1% Tween-20). His-tagged neuropilin-1 b1b2 domain (NRP-1 WT) and triple mutant NS346A-E348A-T349A neuropilin-1 b1b2 domain (NRP-1 mutant) were expressed and purified at the Protein Production and Analysis Facility at the Sanford Burnham Prebys Medical Discovery Institute (La Jolla, CA) as described previously.[10] The recombinant proteins NRP1 WT, NRP1 mutant, and DCN-tCRK were FAM (5-(and-6)-Carboxyfluorescein, #90024, Biotium Inc, CA, USA) labeled by mixing 1:10 ratio of amine-reactive FAM dye (diluted in DMSO final concentration 0.2%) and protein. The mixture reaction was incubated in the dark for 2 hours at RT, followed by ultrafiltration/dialysis with PBS to separate free dye from the protein. 100 µl of FAM-labeled NRP1 WT or NRP1 mutant protein in blocking solution was added to each well (20 µg/well), incubated at room temperature for 4-6 hours at room temperature or 4 °C overnight, and washed 3 times with blocking solution. After adding 100 µl PBS in each well, the plate was immediately read with top read mode using a fluorescence reader (Flex Station II, Molecular Devices; peak excitation = 485 nm, peak emission =530 nm, cut off =515).

For the binding of FAM-DCN-tCRK to NRP-1 positive prostate carcinoma-3 (PC-3) cells (gift from the Ruoslahti laboratory at Sanford-Burnham-Prebys Medical Discovery Institute, La Jolla, CA) and negative melanoma (M21) cells (gift from David Cheresh Lab at University of California San Diego, La Jolla, CA) *in vitro*, the cells were first cultured in growth medium composed of 10% fetal bovine serum (FBS) in DMEM high glucose medium supplemented with penicillin, and streptomycin (Gibco). For experiments, the medium was aspirated, the cells were washed twice with warm medium, and fresh medium was added along with 10 µg FAM-labeled DCN-tCRK recombinant protein. The labelling was done by directly coupling DCN-tCRK recombinant protein to Fluorescein using Lightning-Link Fluorescein kit (Expedon Ltd, UK) according to the manufacturer protocol. The cells were incubated at 37 °C for one hour; medium was aspirated, the cells were washed and fixed with −20 °C methanol. The cells were washed with PBS and blocked (PBS, 1% BSA, 1% FBS, 1% goat serum, 0.05% Tween-20) for 30 minutes at RT followed by primary anti-FITC (Invitrogen, CA, USA. Catalog # A-889) for one hour at RT. The cells were washed, and secondary antibodies Alexa Fluor 488 goat anti-rabbit IgG (Invitrogen, USA) were applied for one hour at RT in the dark. The nuclei of cells were stained with DAPI. The coverslips were mounted on glass slides with Fluoromount-G (Electron Microscopy Sciences, PA, USA), imaged using confocal microscopy (Olympus FV1200MPE, Tokyo, Japan) and analyzed using the FV10-ASW4.2 viewer.

Recombinant protein pharmacokinetics

Recombinant proteins DCN-tCRK or DCN were diluted in Tris buffered saline (TBS) containing 0.05 % Tween-20. The pharmacokinetics of DCN-tCRK and DCN were studied with 8 week old Balb/c male mice. 5 mg/kg either DCN-tCRK or DCN was injected in tail vein under isoflurane anesthesia. Blood samples from distinct tail vein were gathered at 15 min, 30 min, 60 min, 2 h, 4 h, and 16 h after injection. At 8 h or 24 h after the injection, the mice were sacrificed under medetomidine-ketamine anesthesia and blood samples were collected from the subclavian vein. The samples were mixed with 1 M ethylenediaminetetraacetic acid (EDTA), centrifuged 2000 g for 10 min at room temperature and the plasma was stored for analysis. The concentration of human origin decorin in the plasma samples was determined with Human Decorin DuoSet ELISA kit (#DY143, R&D Systems, Minneapolis, MN) according to instructions provided by the manufactured. A venous blood sample from an uninjected mouse was used in each plate to ensure the specificity of the primary antibody.

Collagen lattice contraction assay

Human normal fibroblasts and RDEB patient-derived fibroblasts were cultured in DMEM supplemented with 10% FBS, as previously described.[11] The collagen lattices were prepared by mixing the cell suspension with neutralized rat tail collage type I (Advance BioMatrix, Carlsbad, CA). The final concentration of collagen was 2.4 mg/ml with a cell density of 2.1×10^5 cells/ml. 500 µl of cells/collagen suspension was dispensed into a single well of 24-well plate and

allowed to solidify for 30 min at room temperature. 0.5 ml of DMEM supplemented with 5% of FBS was added in each well after collagen polymerization and plates were cultured at 37ºC with 5% CO_2. After 12 hours of incubation, the gel from each well was gently released by the thin pipet tip and DCN or DCN-CRK were added respectively at a final concentration 75 µM (n=3 per condition). Images were acquired at 12 hours (initial area) and 48 hours (contraction area) respectively and the areas of gels were quantitated using Image J.

Supplemental references

1. Y. Liao, L. Ivanova, R. Sivalenka, T. Plumer, H. Zhu, X. Zhang, *et al.* (2018). Efficacy of Human Placental-Derived Stem Cells in Collagen VII Knockout (Recessive Dystrophic Epidermolysis Bullosa) Animal Model. *Stem Cells Transl Med* **7**: 530-542.
2. T. A. Järvinen (2012). Design of target-seeking antifibrotic compounds. *Methods Enzymol* **509**: 243-261.
3. T. Urakami, T. A. Jarvinen, M. Toba, J. Sawada, N. Ambalavanan, D. Mann, *et al.* (2011). Peptide-directed highly selective targeting of pulmonary arterial hypertension. *Am J Pathol* **178**: 2489-2495.
4. J. H. Park, G. von Maltzahn, L. Zhang, M. P. Schwartz, E. Ruoslahti, S. N. Bhatia, *et al.* (2008). Magnetic Iron Oxide Nanoworms for Tumor Targeting and Imaging. *Adv Mater* **20**: 1630-1635.
5. T. Krusius and E. Ruoslahti (1986). Primary structure of an extracellular matrix proteoglycan core protein deduced from cloned cDNA. *Proc Natl Acad Sci U S A* **83**: 7683-7687.
6. S. Hobbs, S. Jitrapakdee and J. C. Wallace (1998). Development of a bicistronic vector driven by the human polypeptide chain elongation factor 1alpha promoter for creation of stable mammalian cell lines that express very high levels of recombinant proteins. *Biochem Biophys Res Commun* **252**: 368-372.
7. H. E. Klock and S. A. Lesley (2009). The Polymerase Incomplete Primer Extension (PIPE) method applied to high-throughput cloning and site-directed mutagenesis. *Methods Mol Biol* **498**: 91-103.
8. P. G. Scott, P. A. McEwan, C. M. Dodd, E. M. Bergmann, P. N. Bishop and J. Bella (2004). Crystal structure of the dimeric protein core of decorin, the archetypal small leucine-rich repeat proteoglycan. *Proc Natl Acad Sci U S A* **101**: 15633-15638.
9. M. Vähätupa, J. Nättinen, A. Jylhä, U. Aapola, M. Kataja, P. Koobi, *et al.* (2018). SWATH-MS Proteomic Analysis of Oxygen-Induced Retinopathy Reveals Novel Potential Therapeutic Targets. *Invest Ophthalmol Vis Sci* **59**: 3294-3306.
10. T. Teesalu, K. N. Sugahara, V. R. Kotamraju and E. Ruoslahti (2009). C-end rule peptides mediate neuropilin-1-dependent cell, vascular, and tissue penetration. *Proc Natl Acad Sci U S A* **106:** 16157-16162
11. Y. Liao, L. Ivanova, H. Zhu, T. Plumer, C. Hamby, B. Mehta, *et al.* (2018). Cord Blood-Derived Stem Cells Suppress Fibrosis and May Prevent Malignant Progression in Recessive Dystrophic Epidermolysis Bullosa. *Stem Cells* **36**: 1839-1850.
