# Supplementary material for: Exposed CendR Domain in Homing Peptide Yields Skin-Targeted Therapeutic in Epidermolysis Bullosa
Source: Mol Ther. 2020 May 20;28(8):1833–45. doi: 10.1016/j.ymthe.2020.05.017 (PMC7403337; doi:10.1016/j.ymthe.2020.05.017)
Supplement: Document S2. Article plus Supplemental Information [file mmc2.pdf]

Molecular Therapy
Original Article

Exposed CendR Domain in Homing Peptide Yields Skin-Targeted Therapeutic in Epidermolysis Bullosa

Toini Pemmari,[1,11] Larisa Ivanova,[2,11] Ulrike May,[1] Prakash Lingasamy,[3] Allan Tobi,[3] Anja Pasternack,[4] Stuart Prince,[1] Olli Ritvos,[4] Shreya Makkapati,[2] Tambet Teesalu,[3,5,6] Mitchell S. Cairo,[2,7,8,9,10] Tero A.H. Järvinen,[1,11] and Yanling Liao[2,11]

[1]Faculty of Medicine and Health Technology, Tampere University & Tampere University Hospital, 33520 Tampere, Finland; [2]Department of Pediatrics, New York Medical College, Valhalla, NY 10595, USA; [3]Laboratory of Cancer Biology, Institute of Biomedicine and Translational Medicine, University of Tartu, 50411 Tartu, Estonia; [4]Department of Physiology, Faculty of Medicine, University of Helsinki, 00014 Helsinki, Finland; [5]Cancer Center, Sanford Burnham Prebys Medical Discovery Institute, 10901 North Torrey Pines Road, La Jolla, CA 92037, USA; [6]Center for Nanomedicine, University of California, Santa Barbara, CA 93106, USA; [7]Department of Microbiology and Immunology, New York Medical College, Valhalla, NY 10595, USA; [8]Department of Pathology, New York Medical College, Valhalla, NY 10595, USA; [9]Department of Medicine, New York Medical College, Valhalla, NY 10595, USA; [10]Deparmtent of Cell Biology and Anatomy, New York Medical College, Valhalla, NY 10595, USA

Systemic skin-selective therapeutics would be a major advancement in the treatment of diseases affecting the entire skin, such as recessive dystrophic epidermolysis bullosa (RDEB), which is caused by mutations in the COL7A1 gene and manifests in transforming growth factor-β (TGF-β)-driven fibrosis and malignant transformation. Homing peptides containing a C-terminal R/KXXR/K motif (C-end rule [CendR] sequence) activate an extravasation and tissue penetration pathway for tumor-specific drug delivery. We have previously described a homing peptide CRKDKC (CRK) that contains a cryptic CendR motif and homes to angiogenic blood vessels in wounds and tumors, but it cannot penetrate cells or tissues. In this study, we demonstrate that removal of the cysteine from CRK to expose the CendR sequence confers the peptide novel ability to home to normal skin. Fusion of the truncated CRK (tCRK) peptide to the C terminus of an extracellular matrix protein decorin (DCN), a natural TGF-β inhibitor, resulted in a skin-homing therapeutic molecule (DCN-tCRK). Systemic DCN-tCRK administration in RDEB mice led to inhibition of TGF-β signaling in the skin and significant improvement in the survival of RDEB mice. These results suggest that DCN-tCRK has the potential to be utilized as a novel therapeutic compound for the treatment of dermatological diseases such as RDEB.

INTRODUCTION

A general limitation in systemic drug delivery is that only a small fraction of drug reaches its desired location and systemic side effects are encountered in other organs. Thus, a critical goal of modern drug development is to generate drugs to be target organ-specific, with minimal adverse effects in the other parts of the body. This goal could be achieved by developing drugs that recognize a specific epitope expressed in the affected organ. Alternatively, drugs can be converted to be target-specific by conjugation with an affinity ligand such as a homing peptide.[1–3] *In vivo* screening of phage peptide libraries has identified organ- or disease-specific molecular signatures in the vascular tissues, enabling a postal code system (vascular zip codes) for target-specific delivery of systemically administered therapeutics.[2–4] The most efficient vascular homing peptides for tumor-specific cell and tissue penetration contain a consensus motif R/KXXR/K, with an arginine (or rarely lysine) residue at the C terminus, thus called a C-end rule (CendR) sequence.[5–8] The CendR sequence binds to neuropilin-1 (NRP-1), activating an extravasation and tissue penetration pathway that delivers the peptide along with its payload into the parenchyma of the tumor tissue.[3,5,8] As NRP-1 is expressed by the endothelial cells in all tissues,[3] peptides containing cryptic CendR owe their target selectivity to a combination of binding to primary receptor with a tumor-specific expression pattern, and to a proteolytic activation to expose the CendR sequence in the target organ.[5–8]

Being the largest organ of the human body, skin presents unique challenges for efficient drug delivery. The primary challenge related to local, i.e., transdermal, drug delivery is the poor penetration of macromolecules into the skin. Diffusion through intercellular lipids

Received 3 October 2019; accepted 14 May 2020;
https://doi.org/10.1016/j.ymthe.2020.05.017.

[11]These authors contributed equally to this work.

Correspondence: Yanling Liao, PhD, Department of Pediatrics, New York Medical College, Valhalla, NY 10595, USA.
E-mail: yanling_liao@nymc.edu

Correspondence: Tero A.H. Järvinen, MD, PhD, Faculty of Medicine and Health Technology, Tampere University & Tampere University Hospital, 33520 Tampere, Finland.
E-mail: tero.jarvinen@tuni.fi

This is an open access article under the CC BY license (http://creativecommons.org/licenses/by/4.0/).

provides a transdermal delivery option, but it is limited only for the delivery of small lipophilic molecules. Therefore, systemically administered, yet skin-specific therapeutics would be a substantial therapeutic advance for the treatment of skin diseases, particularly those that affect the entire skin, such as recessive dystrophic epidermolysis bullosa (RDEB). RDEB is caused by mutations in the COL7A1 gene that encodes type VII collagen (C7).[9–12] Clinical manifestations include skin erosions and blistering, mutilating scarring, pseudosyndactyly, and a high risk of developing aggressive and rapidly metastasizing cutaneous squamous cell carcinomas (cSCCs).[10,11,13–16] Although novel gene-, cell-, and protein-based therapies have demonstrated promising results in delivering C7 to the skin, challenges remain and there is still no cure for RDEB.[13–16] TGF-β signaling has been demonstrated to play an essential role in the development of fibrosis and in the progression to malignancy in RDEB.[17–19] Our previous study demonstrated that TGF-β signaling is activated as early as a week after birth in *col7a1*$^{-/-}$ mice.[20] Thus, an early intervention on the activation of TGF-β signaling may be beneficial in reducing disease burden in RDEB. TGF-β signaling has also been suggested to be a phenotypic modulator in monozygotic twins with identical COL7A1 mutations.[18] Moreover, the expression level of a proteoglycan decorin (DCN), a natural TGF-β inhibitor, was significantly higher in the less affected twin. DCN is a structural constituent of extracellular matrix (ECM), and *Dcn*$^{-/-}$ mice exhibit irregular collagen fibril formation and significantly reduced tensile strength in skin.[21] Furthermore, DCN has anti-fibrotic and anti-tumor functions by regulating activities of multiple growth factors, among them inhibitory action on TGF-β.[22,23] We recently also demonstrated an upregulation of DCN expression as one of the mechanisms of action for the effects of cord blood-derived unrestricted somatic stem cells (USSCs) in *col7a1*$^{-/-}$ mice.[20] Supporting the role of DCN as a potential therapeutic disease-modifying molecule for RDEB, Cianfarani et al.[24] recently reported that systemic administration of lentivirus driving the expression of human DCN (hDCN) attenuated TGF-β-induced fibrosis in a C7-hypomorphic RDEB mouse model that expresses a residual level of C7 (C7-hypomorphic mice).

Our past *in vivo* phage display screens identified a panel of peptides that home to angiogenic blood vessels in skin wounds.[25] Two of the most promising peptides, cyclic peptides dubbed CAR (CARSKNKDC) and CRK (CRKDKC), have been utilized to deliver different therapeutic molecules in a target-selective fashion.[1] Interestingly, whereas CRK peptide contains a cryptic CendR sequence, RKDK, it is the only peptide among the vascular-homing CendR peptides that is not capable of penetrating cells and tissues.[25,26] In this study, we demonstrate that C-terminal exposure of the cryptic CendR-sequence of CRK, i.e., truncated CRK (tCRK; CRKDK), confers the peptide the ability to home to and penetrate normal skin while retaining its ability to home to skin wounds. This novel targeting specificity can be used for therapeutic benefit; that is, recombinant DCN-tCRK fusion protein had a superior therapeutic effect compared to native DCN in a *col7a1*$^{-/-}$ RDEB mouse model that completely lacks expression of C7.

RESULTS

tCRK Peptide Homes to Skin Wounds and Normal Skin

We previously characterized the homing of CAR and CRK peptides to skin wounds at different phases of wound healing.[25] As CRK contains a cryptic CendR motif, but is not capable of penetrating cells and tissues,[25,26] we set out to investigate whether truncation of CRK by removing the last cysteine residue to expose the CendR motif at the C terminus would change its homing and/or tissue-penetration properties.

We first determined the homing of tCRK to wounded skin at day 7 at the peak of angiogenesis. Similar to our previous report on CAR and CRK,[25] the phage carrying tCRK peptide homed to the wound 112 ± 71.2-fold higher than the nonrecombinant control phage ($p < 0.001$, $n = 8$) (Figure 1A). The wound homing of tCRK was not statistically different from phage displaying CAR peptide (101 ± 89.3-fold higher than negative control, $n = 5$), but it was significantly better than the CRK phage (15.5 ± 8.19-fold, $p < 0.05$, $n = 8$) (Figure 1A). Our previous studies also showed that CAR, but not CRK, homes to early angiogenic blood vessel sprouts in day 5 wounds.[25] In this study, we observed that tCRK also homes to the day 5 wound at a significantly higher level than the nonrecombinant phage (6.14 ± 1.41-fold, $p < 0.05$, $n = 9$) (Figure S1).

As wound healing progresses, the granulation tissue rich in angiogenic blood vessels gradually converts to scar tissue with a limited number of blood vessels. The homing of CRK and CAR were diminished to 13.6 ± 2.66-fold ($p < 0.001$, $n = 7$) and 20.7 ± 4.72-fold ($p < 0.001$, $n = 7$) when compared to the control phage at the excisional wound on day 14 (Figure 1B). In contrast, there was an ~35-fold enrichment of tCRK peptide at this time point (34.3 ± 9.78-fold, $p < 0.001$, $n = 7$). These results show that tCRK homes to skin wounds at different phases of wound healing and is able to home to wounds that have matured to scar tissue.

There was no overrepresentation of the phages displaying tCRK, CAR, or CRK peptides at other organs, including lung, heart, spleen, kidney, and liver (Figure 1C). Surprisingly, in addition to homing to the wound, tCRK was also detected in normal skin, 3 cm or farther from the edge of the wound (Figure 1C), and the enrichment (11.2 ± 9.16-fold) was remarkably higher than any other studied phage clones. To exclude possible influence of a nearby wound on the tCRK homing to normal skin, we investigated the skin homing property of tCRK in mice without wounds. Indeed, about 20-fold (20.5 ± 5.67-fold, $p < 0.001$, $n = 7$) enrichment of tCRK compared to the control phage was seen in the normal skin (Figure 1D). This was significantly higher ($p < 0.05$) than that of CRK (5.04 ± 2.88-fold).

To confirm that tCRK peptide indeed homes to both normal skin and skin wounds, we generated nanoparticles, iron oxide nanoworms (IONWs), and coupled them with/without tCRK-homing peptides. IONWs were synthesized and characterized as described previously.[27] Fluorescent tCRK, a scrambled CendR peptide (negative control

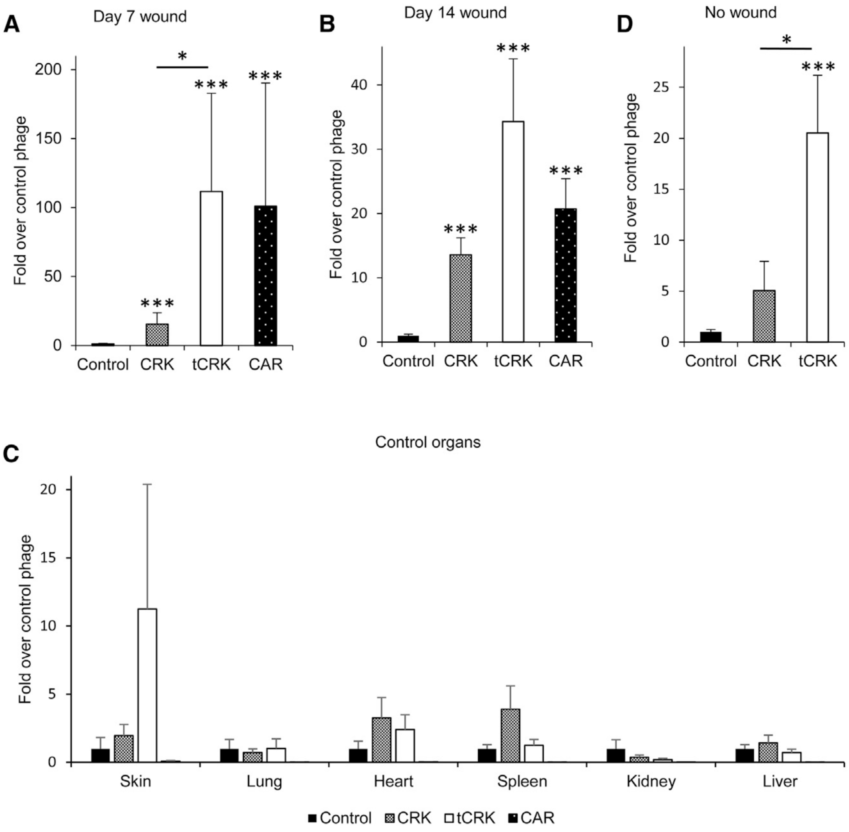

Figure 1. Homing of tCRK Phage

The amounts are represented as fold over control phage. (A) Phage homing in 7-day-old wound. n = 8 for tCRK and CRK, n = 5 for CAR, and n = 7 for control phage. (B) Phage homing in 14-day-old wound. n = 7 for tCRK, CRK, and CAR, and n = 5 for control phage. (C) Phage homing in control organs. n = 11 for tCRK and control phage, n = 10 for CRK, and n = 5 for CAR. (D) Phage homing in unwounded skin. n = 7 for all phages. Error bars represent SEM. *$p < 0.05$, **$p < 0.01$, ***$p < 0.001$, Kruskal-Wallis test with Bonferroni *post hoc* test.

dubbed PRP), or FAM label alone was coated on the IONWs through a thioether bond between the cysteine thiol from the peptide and the maleimide on the IONWs.[28] The IONWs were injected into mice with either excision wounds with splints or without splints. Only tCRK-coated IONWs, but not control peptide or FAM-coated IONWs, were detected throughout the blood vessels of the normal dermis (taken farther than 5 cm from the wounds) (Figure 2). In both excision wound models, strong accumulation of tCRK-coated IONWs was detected in hypervascular regions of the wounds, whereas these hypervascular regions were almost devoid of control peptide or FAM-coated IONWs (Figure S2). These results further confirmed that exposure of cryptic CendR sequence facilitates tCRK to be not only a potent wound-homing peptide but also a peptide homing to normal skin.

Generation of Multi-Functional, Recombinant DCN-tCRK Fusion Protein

We next engineered DCN-tCRK fusion protein by placing tCRK peptide at the C terminus of DCN (Figure 3A). Both DCN-tCRK and native DCN were expressed in mammalian cells and purified using chromatography (Figure S3A). Both recombinant proteins migrated as sharp bands at about 55 kDa with a smear above the band in SDS gel electrophoresis and detected as DCN by western blot analysis (Figure S3B). The sharp band corresponds to the core protein, and the smear is caused by heterogeneity in the glycosaminoglycan sulfate chain (mostly chondroitin) attached to the DCN core. Mass spectrometry validated the identity of DCN and the C-terminal tCRK sequence (Table S1). The hydrodynamic size indicates that DCN-tCRK exists as homogeneous and non-aggregated macromolecules with a diameter consistent with the reported dimer of DCN[29] (Figure S3C). Differential scanning calorimetry produced a sharp peak with a melting temperature (Tm) of 49°C, suggesting that tCRK-DCN will maintain a stable tertiary structure at a physiological condition (Figure S3D).

DCN-tCRK Interacts with NRP-1 *In Vitro*

We next investigated whether the tCRK peptide fused to DCN retains its ability to interact with NRP-1. We immobilized DCN-tCRK on ELISA plates and tested its binding to wild-type (WT) or mutant NRP-1, where the CendR-binding pocket was disabled by a triple mutation.[6] DCN-tCRK effectively binds to WT NRP-1 at a significantly higher level than the control bovine serum albumin (BSA) ($p < 0.01$), whereas it showed no significant binding to the mutant NRP-1 ($p > 0.05$) (Figure 3B). Furthermore, parallel studies with a synthetic RPARPAR peptide, a prototypic CendR peptide, and RPARPARA, a control peptide with a C-terminally capped CendR sequence and unable to interact with NRP-1, were used to fortify that the binding is dependent on CendR sequence (Figure 3B). We further determined whether DCN-tCRK binds to the cells that express NRP-1, i.e., human PC3 prostate carcinoma cells. M21 melanoma cells that do not express NRP-1 were also included in the assay. Supporting the NRP-1-dependent cell binding and penetration properties, internalization of DCN-tCRK was observed only in the NRP-1-positive PC3 cells, but not in the NRP-1-negative M21 cells (Figure 3C).

DCN-tCRK and DCN Exhibited Similar Pharmacokinetics *In Vivo*

To determine whether the addition of tCRK peptide had any effect on the circulation half-life of DCN, DCN-tCRK and DCN were injected intravenously in parallel in healthy BALB/c mice, and their amount in peripheral blood at different time points within 24 h of administration was quantitated by ELISA. The half-life of DCN-tCRK in blood was 30 min and was not significantly different from that of DCN (Figure S4). The pharmacokinetic studies suggest that modification of

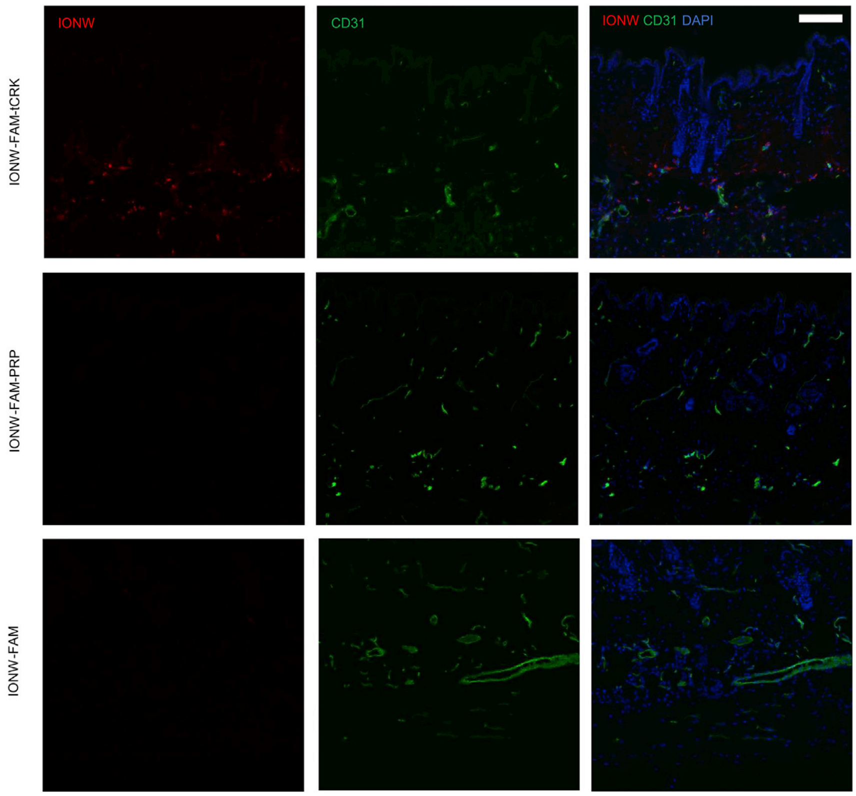

Figure 2. Homing of tCRK-Coated Nanoparticles to Normal Skin

Representative images of immunohistochemical staining of skin samples from BALB/c mice with anti-FITC (fluorescein isothiocyanate) (red) to detect the intravenously (i.v.) injected FAM-labeled IONWs coated with tCRK (12 mg/kg), a control peptide (PRP) (16 mg/kg), or no peptide (13 mg/kg). The localization of blood vessels is depicted by anti-CD31 (green), and nuclei are stained with DAPI (blue). Scale bar, 100 μm. Representative images are from three independent experiments.

DCN with small vascular-homing peptide does not influence the pharmacokinetics of DCN.

DCN-tCRK Administration Improves the Survival of *col7a1*$^{-/-}$ Mice

We next evaluated the therapeutic function and skin-homing properties of DCN and DCN-tCRK in *col7a1*$^{-/-}$ mice, an animal model of RDEB. These mice are generated by breeding of the heterozygous littermates, and *col7a1*$^{-/-}$ mice can be identified at birth based on the manifestation of hemorrhagic blistering in the skin.[30] The newborn *col7a1*$^{-/-}$ mice were randomly divided to receive DCN, DCN-tCRK, or PBS (negative control) via intrahepatic administration. Repeated intraperitoneal (i.p.) administration was performed to the surviving mice within each group every other day after the first dose until day 14. In this study, the median lifespan of *col7a1*$^{-/-}$ mice was 2 days after PBS injection and it was significantly prolonged to 7 days after administration of DCN ($p < 0.0001$) (Figure 4A). However, the survival of *col7a1*$^{-/-}$ mice after DCN administration was not statistically significant as compared to a historical administration of dextran/human serum albumin (D/HSA), which was used as the vehicle for stem cell administration and sporadically increased the survival of some *col7a1*$^{-/-}$ recipient mice likely by adjusting fluid balance (Figure S5).[31] Moreover, DCN injections did not extend the survival of the recipients beyond 2 weeks of age. Importantly, the median lifespan of the mice after DCN-tCRK treatment was further extended to 11 days, which was significantly better than that after either PBS ($p < 0.0001$) or historical D/HSA administration ($p < 0.001$) (Figures 4A and S5). In addition, 85% of DCN-tCRK-treated mice reached 7 days of survival, and 20% of these mice survived past 3 weeks of age and were subsequently sacrificed for skin analyses.

DCN-tCRK Homes in Skin of *col7a1*$^{-/-}$ Mice

We next utilized an ELISA assay to quantitate hDCN and DCN-tCRK in the skin of recipient RDEB mice at 1, 2, and 3 weeks ($n = 3$ for all time points) (Figure 4B). There was no statistically significant difference between DCN-tCRK- and DCN-treated skin at the 1-week time point. However, the level of DCN-tCRK at the 2-week time point was significantly higher than that of DCN (3.6-fold, $p < 0.05$) (Figure 4B). In addition, as the last i.p. administration of DCN-tCRK was conducted on day 14, identification of DCN-tCRK in the 3-week skin (19.47 ± 12.80 pg/mL) is highly suggestive of its stability *in vivo* for at least 7 days.

We also performed immunohistochemical staining based on the expression of histidine tag to analyze the anatomical distribution of DCN-tCRK or DCN in the RDEB skin. DCN-tCRK was detected in the dermis of both the paw and dorsal skin of the RDEB mice at 1, 2, and 3 weeks (Figure 4C). Moreover, staining of the gastrointestinal (GI) tract of the recipient RDEB mice did not reveal reactivity with anti-his antibody (data not shown), suggestive of a skin-specific targeting of DCN-tCRK. In contrast, although ELISA demonstrated the presence of DCN in the skin lysate, the anti-his immunostaining on DCN-treated RDEB skin, represented by the 1-week time point, only appeared to be non-specific (diffuse) (Figure 4C). Further supporting our hypothesis that the homing of DCN-tCRK is afforded by NRP-1-dependent cell and tissue penetration, the anti-his and anti-NRP-1 double staining demonstrated that the signal from DCN-tCRK was within or in a close proximity to the cells that were positive for NRP-1 in RDEB skin (Figure 4D).

DCN-tCRK Therapy Suppresses the Fibrotic Responses in RDEB Mice

Our recent studies demonstrated a significant elevation of TGF-β signaling in *col7a1*$^{-/-}$ mice beginning in the interdigital folds of

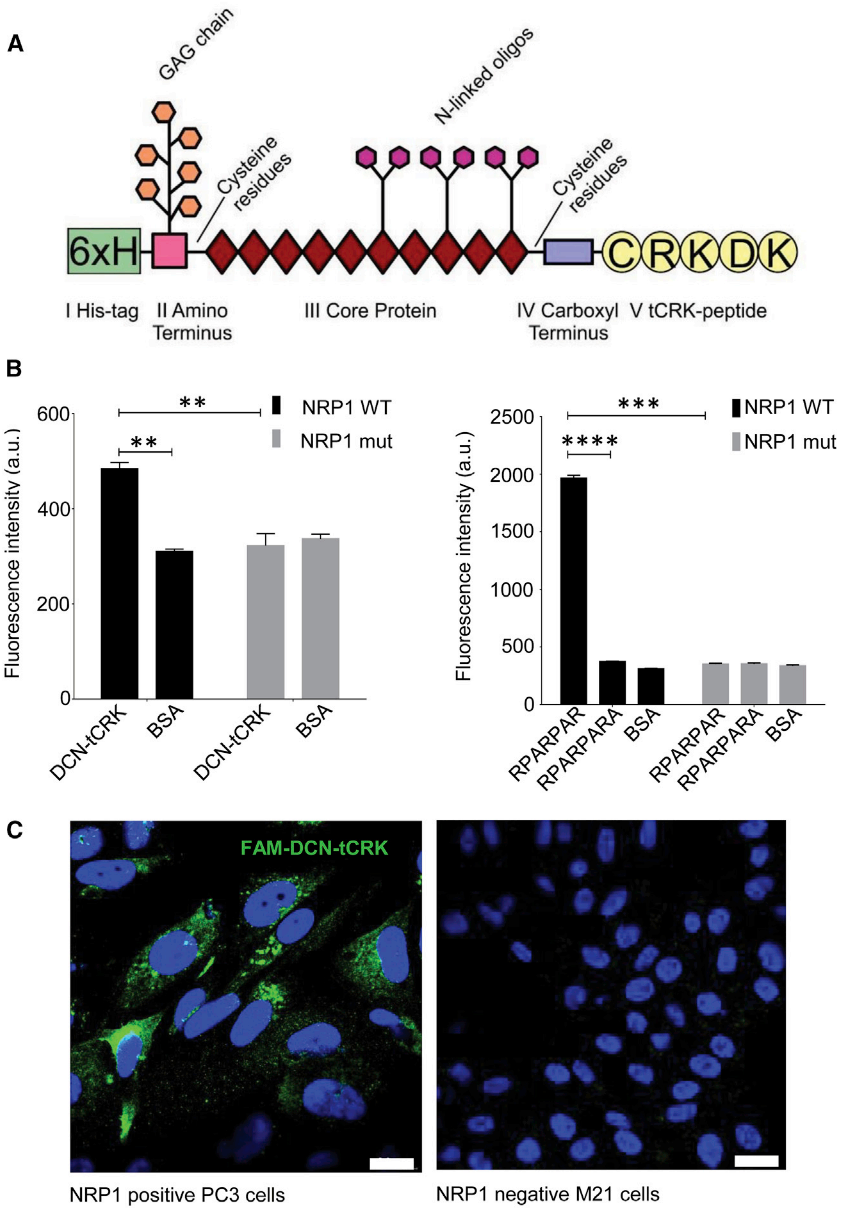

Figure 3. Recombinant DCN-tCRK Protein and Its Binding to Neuropilin-1

(A) A schematic representation of the structure of DCN-tCRK. Signal peptide and propeptide of the native DCN were replaced with a 6× His-tag (I) for purification. The His-tag is followed by the amino terminus (II), core protein (III), and carboxyl terminus (IV) of mature DCN proteoglycan. tCRK peptide (V) was cloned on the carboxyl end of the protein. (B) *In vitro* binding of DCN-tCRK to neuropilin-1 (NRP-1) *in vitro*. DCN-tCRK (left panel) and peptide controls (right panel, positive peptide [RPARPAR] and negative peptide [RPARPARA]) were immobilized in ELISA plate. Bovine serum albumin (BSA) was included as a non-specific protein control for DCN-tCRK and the peptides. WT and mutant NRP-1 were labeled with FAM and added to the immobilized plate. The binding of the NRP-1 was measured based on fluorescence intensity. Error bars represent SEM. Experiments were repeated with triplicate samples. **p < 0.01, ***p < 0.001, ****p < 0.0001, Student's unpaired t test. (C) Internalization of DCN-tCRK in the NRP-1-positive cells. FAM-labeled DCN-tCRK was incubated with PC3 and M21 cells positive and negative for NRP-1 expression, respectively. DCN-tCRK was detected by anti-FAM immunostaining (green). Nuclei were counterstained with DAPI (blue). Representative images from three independently studied experiments are shown. Scale bars, 20 μm.

the paws as early as a week after birth.[20] Therefore, in this study, we chose the skin biopsies of this time point to compare the expression of 84 genes central to wound-healing responses and fibrosis formation between the WT and vehicle (D/HSA)-, DCN-, or DCN-tCRK-treated RDEB skin (n = 3 per group) (Table S2). Relative to the WT, more than half of the genes showed a >1.5-fold increase in expression in the vehicle-injected RDEB skin, as demonstrated in the clustergram in Figure 5A. The relative fold changes ($\log_2$) of gene expression and the p values ($-\log_{10}$) are also presented as volcano plots, and the significantly ($p < 0.05$) dysregulated genes are marked in red in each plot (Figure 5B). The significantly upregulated genes in the vehicle RDEB skin are involved in TGF-β signaling (i.e., *Tgfb1*, *Tgfbr3*, *Ctgf*), WNT signaling (*Ctnnb1*), mitogen-activated protein kinase (MAPK)1/MAPK3 signaling (*Mapk3*), and epidermal growth factor receptor (*Egfr*) signaling, ECM remodeling (*Ctsg*, *Plaur*), cell adhesion (*Itgb3*, *Itgb5*), and inflammation (*Il4*, *Cxcl3*, *Tnfα*). There were no significantly downregulated genes in the vehicle RDEB skin compared to the WT. In the DCN-treated RDEB mouse skin, the overall gene expression profile was similar to that in the vehicle RDEB skin (Figure 5B). Even though the expression of *Tgfb1*was no longer significantly abnormal, the expression of *Tgfbr3* and *Ctgf* was still significantly upregulated in the DCN-treated RDEB skin. Some genes, such as *Il4*, *Cxcl3*, and *Tnfα*, were more significantly upregulated in the DCN-treated RDEB skin than in the vehicle control (Figure 5B; Table S2).

Importantly, the expression profile of DCN-tCRK-treated RDEB skin was markedly different from those of vehicle- and DCN-treated RDEB skin and resembled that of WT skin (Figure 5A). Although it showed individual variation in the expression of some genes, none of the genes in the array was significantly dysregulated in DCN-tCRK-treated RDEB skin when compared to the WT (Figure 5; Table S2).

Supporting the development of TGF-β1-mediated fibrosis in untreated RDEB skin and its suppression by DCN-tCRK treatment,

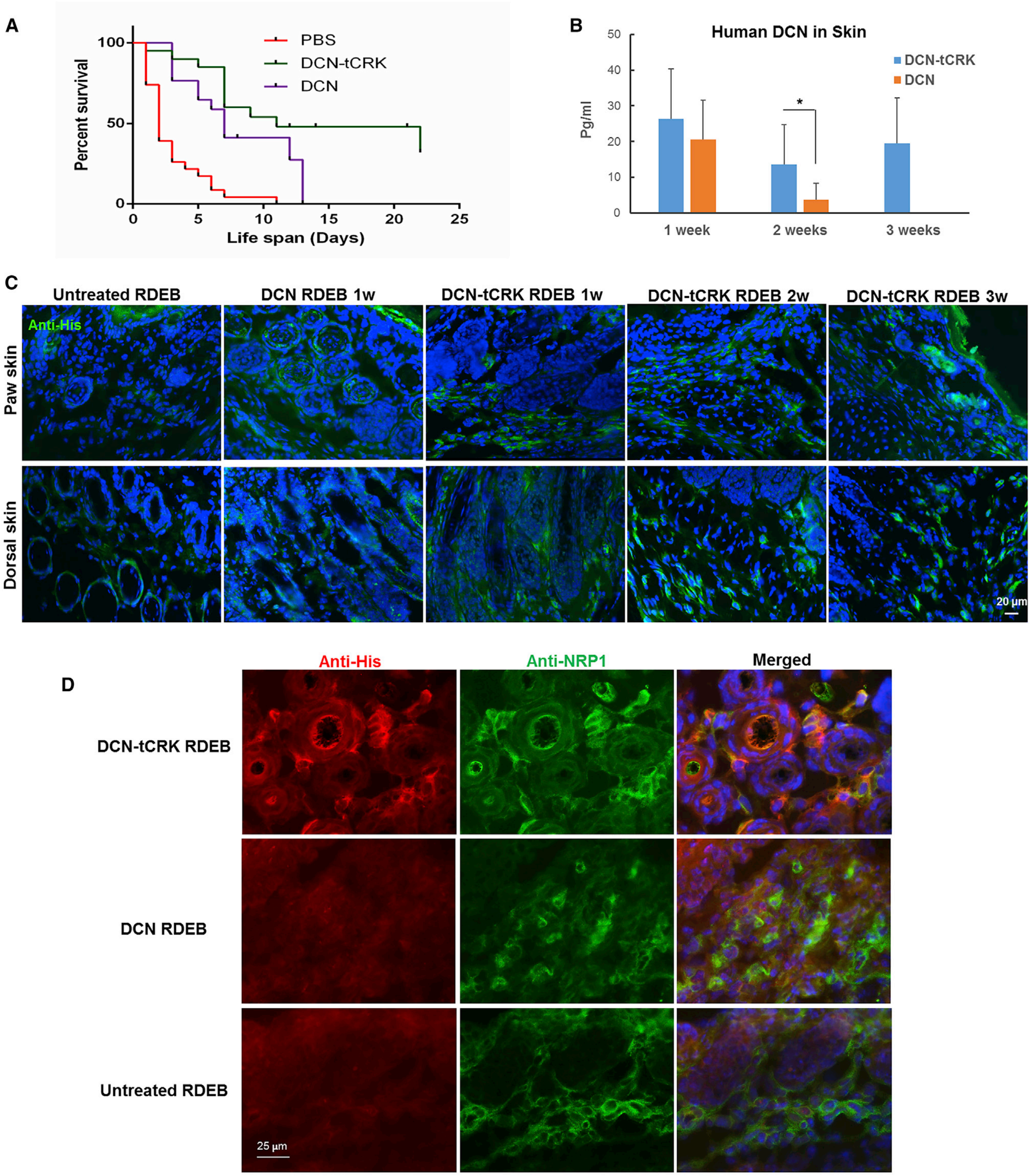

Figure 4. DCN-tCRK Improves Survival of *col7a1*$^{-/-}$ Mice and Homes to the Skin

(A) Kaplan-Meier survival analysis of the *col7a1*$^{-/-}$ mice that received DCN-tCRK (median lifespan, 11 days; n = 21; green line), DCN (median lifespan, 7 days; n = 17; purple line), and PBS (median lifespan, 2 days; n = 24; red line) administration. (B) Quantitation on the levels of DCN and DCN-tCRK, determined using a human decorin ELISA kit, in the skin of recipient *col7a1*$^{-/-}$ mice at 1 week, 2 weeks, and 3 weeks after intrahepatic administration (n = 3 per time point). There was no quantitation on the level of DCN at the 3-week time point, as no mice survived until that time after DCN administration. Error bars represent SEM. *$p < 0.05$, **$p < 0.01$. (C) Immunohistochemical staining using

(legend continued on next page)

strong expression of CTGF/CCN2 was observed in vehicle-injected RDEB skin, and the expression level was markedly diminished after treatment with DCN-tCRK (Figure 6A). Moreover, the overall collagen deposition increased with time in the vehicle-injected RDEB skin, as demonstrated by picrosirius red staining, but was significantly decreased in the DCN-tCRK-treated mouse skin (Figures 6B and 6C). Immunostaining demonstrated a substantial increase in the expression of type I collagen (COL1) in the vehicle-treated skin and an attenuated expression in the DCN-tCRK-treated skin at the 2-week time point (Figures 6D and 6E). Similar results were obtained with immunostaining of myofibroblasts, i.e., α-smooth muscle actin (αSMA; Figures 6D and 6E). Moreover, most of the αSMA^{+} cells in the WT as well as DCN-tCRK-treated RDEB skin co-localized with blood vessels (CD31 staining), which indicates their identity as blood vessel smooth muscle cells and pericytes, whereas the αSMA^{+} cells in the vehicle-treated RDEB skin were outside of the blood vessels, i.e., indicative of being myofibroblasts (Figure 6D).

To directly demonstrate the anti-fibrotic function of DCN-tCRK, we compared the abilities of DCN and DCN-tCRK to suppress the collagen gel contraction *in vitro*, using both normal and RDEB-derived fibroblasts. At a low concentration (75 μM), at which DCN had no significant effect on collagen contraction, DCN-tCRK suppressed the collagen gel contraction in both normal ($p < 0.05$) and RDEB-derived ($p < 0.01$) fibroblasts (Figure S6). These findings are in line with our previous report that modification of DCN with a vascular-homing peptide CAR makes the native DCN biologically more active by enhancing its binding to target cells.[23,32]

DISCUSSION

We demonstrate that a C-terminal exposure of CendR sequence in a wound-homing peptide renders a novel tissue-penetrating function of the peptide in normal and wounded skin. Conjugation of tCRK peptide to DCN facilitates skin-selective targeting of the therapeutic fusion protein that exerts anti-fibrotic effects and improves survival in a murine model of RDEB.

Currently, homing peptides containing a CendR motif are under extensive investigation as a novel method to enhance the efficacy of drug delivery in cancer treatment.[2–4] Most CendR peptides, with the exception of tLyP-1,[33] contain cryptic CendR. Cryptic CendR peptides home to tumor vasculature by binding to the receptor expressed selectively on tumor endothelium, where they are cleaved by proteases and the cryptic CendR motif is exposed, after which it can bind to NRP-1 for efficient cell and tissue penetration. None of these peptides home to normal skin. The CendR motif in the tCRK peptide is exposed at the C terminus at the time of systemic administration and does not require proteolytic cleavage before activation of the CendR pathway. Our *in vitro* studies confirmed the binding of tCRK to NRP-1 and cell penetration of tCRK in an NRP-1-dependent manner. In the *in vitro* binding analysis, we used a prototypic peptide with an exposed CendR sequence, i.e., RPARPAR, as a positive control. It is the strongest CendR peptide characterized to date.[6] When injected into the circulation through the tail vein, the RPARPAR peptide binds to NRP-1 in the first vascular bed it meets, i.e., lung, and does not circulate to the systemic side of the circulation.[6] As tCRK is a substantially weaker NRP-1 binding peptide than RPARPAR, tCRK may be able to circumvent NRP-1 in the majority of the tissues. Variable expression levels of NRP-1 in different endothelial populations and/or ideal blood flow conditions may have rendered NRP-1 available for the tCRK binding preferably in skin.

To validate the utility of tCRK as a delivery cargo for skin-targeted therapies, we conjugated tCRK to DCN. In addition to binding and neutralizing all isoforms of TGF-β,[23] DCN also competes with TGF-β receptor 3 (TGFBR3) on TGF-β binding (by having the same binding site).[34,35] As TGFBR3 is an important co-receptor for TGF-β and the binding to it enhances TGF-β signaling, the direct neutralization of TGF-β and the inhibition of TGF-β-TGFBR3 binding by soluble DCN could be one of the mechanisms by which DCN-tCRK exerts its biological effects. Moreover, DCN binds and neutralizes CTGF/CCN2, which is a downstream mediator of TGF-β's fibrotic signaling and has been proposed to be a therapeutic target in the prevention of scarring.[36,37] As the binding sites for TGF-β and CCN2 reside in different parts of DCN, DCN theoretically can simultaneously block both mediators of fibrosis. Indeed, the role of DCN on suppressing TGF-β-driven scar formation has been well established in numerous disease models such as renal, lung, and hepatic fibrosis and in skin wound healing, in addition to RDEB.[18,20,24,25,32,38,39] However, despite numerous positive anti-cancer and anti-fibrotic results in preclinical studies,[22,23] DCN has not reached the clinic as a systemic therapy. So far, the only reported clinical application of DCN was in 12 patients with perforating eye injury, and a single dose of either 200 or 400 μg of human recombinant DCN intravitreal injection appeared to be well tolerated with no ocular adverse events.[40]

We demonstrated that systemic administration of DCN-tCRK recombinant protein was more effective than unmodified DCN in improving the survival of $col7a1^{-/-}$ mice. The exact molecular mechanism is not known, but we assume that multiple different mechanisms could contribute to the improved survival. DCN is an anti-inflammatory and anti-fibrotic molecule. Consistent with our previous finding on the activation of TGF-β signaling as early as a week after birth,[20] the expression levels of more than half of the genes related to fibrosis formation were upregulated in the untreated RDEB mouse skin at the 1-week time point. The improved survival of RDEB mice by DCN-tCRK administration is likely related to the anti-fibrotic and anti-inflammatory effects of the therapeutic protein.

anti-histidine antibody (anti-his; green) on both paw and dorsal skin of $col7a1^{-/-}$ mice is presented. Nuclei were counterstained with DAPI (blue). Scale bar, 20 μm. (D) Representative double staining of anti-histidine tag (red) and anti-NRP-1 (green) and the merged image (with DAPI counterstain; blue) of the DCN-tCRK-treated, DCN-treated, and untreated RDEB skin are presented. Scale bar, 25 μm.

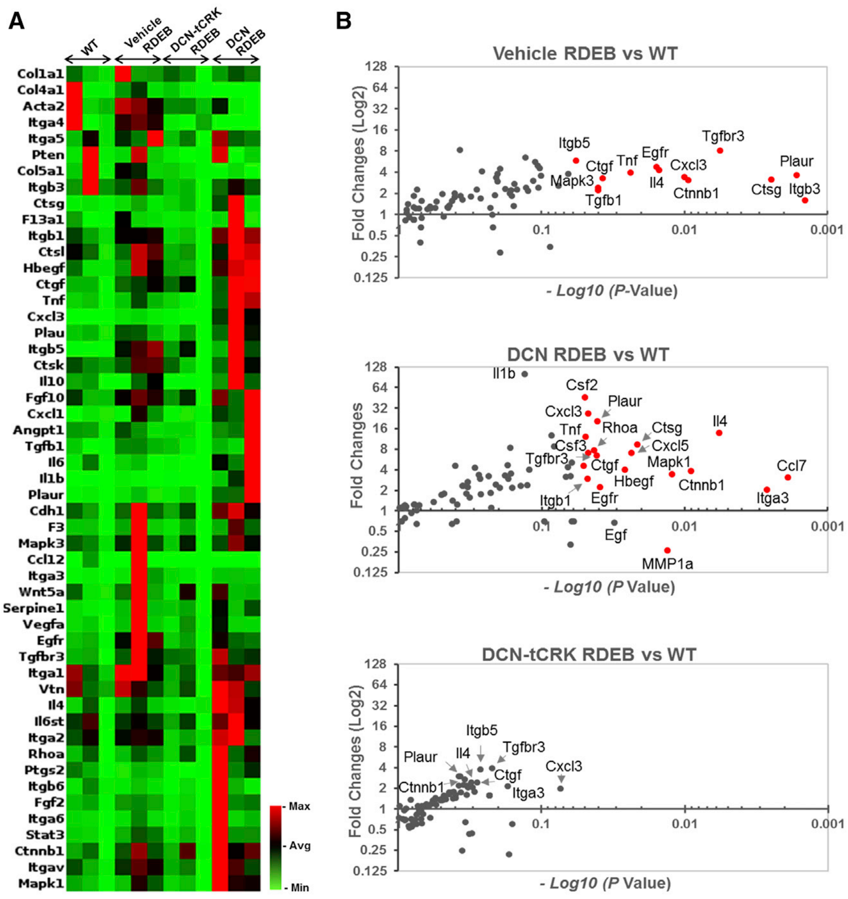

Figure 5. DCN-tCRK Normalizes Fibrotic Gene Signature in RDEB

(A) Relative gene expression in clustergram for the genes that had a >1.5-fold increase in expression in the untreated RDEB skin than for the WT. (B) Volcano plots on $\log_2$ fold changes and $-\log_{10}$ p values of gene expression in the vehicle-, DCN-, and DCN-tCRK-treated *col7a1*$^{-/-}$ mouse skin relative to the WT.

However, this mechanism does not provide a plausible explanation for the improved survival during the first week of life. In this context, it is worth remembering the natural function of DCN. The absence of DCN (*Dcn*$^{-/-}$) leads to fragile skin due to the reduced tensile strength.[41] DCN can bind to the same fibrillar collagens expressed in the dermis, i.e., type I, III, IV, and V collagen,[42] as C7 does, and also similar to C7, DCN is known to connect type VI collagen to fibrillar collagens.[43] Furthermore, the GAG side of DCN, in turn, can bind to multiple ligands, among them integrins, and tCRK peptide affords binding to NRP-1 in DCN-tCRK. Since DCN-tCRK has the versatile ability to bind and connect ECM components, especially different collagens, it could also maintain the mechanical integrity of the skin, which it is heavily compromised in RDEB and thus improve the survival.

Not only were the genes directly involved in TGF-β signaling normalized in the DCN-tCRK-treated (but not in the DCN-treated) RDEB skin, the genes related to other signaling pathways, such as β-catenin and EGFR, were also normalized by DCN-tCRK administration. Both Wnt/β-catenin and EGFR signaling have been demonstrated to contribute to fibrogenesis in multiple fibrotic diseases through their independent, profibrotic mechanisms or via cross-talking with the TGF-β signaling.[37,38,44,45] For example, EGFR activation is required for profibrotic functions of TGF-β[44] and CCN2-mediated fibroblast proliferation and myofibroblast transdifferentiation.[46,47] DCN can bind and downregulate EGFR and HGF receptor Met (to suppress expression of β-catenin).[48,49] The normalized expression of these genes in the *col7a1*$^{-/-}$ mouse skin after administration of DCN-tCRK suggests multiple therapeutic functions of DCN-tCRK in RDEB. The upregulation of pro-inflammatory genes in DCN-treated RDEB skin, in turn, may indicate a therapeutic effect that was not sustained by the administration of native DCN.

When we analyzed skin homing of DCN-tCRK and DCN using ELISA analysis, the amount of DCN in the skin lysate was not statistically different from that of DCN-tCRK at the 1-week time point. DCN core protein has been reported to home to angiogenesis[50,51] and has been used as a delivery vehicle for other therapeutics.[52,53] Most recently, a collagen-binding peptide derived from DCN sequence was a potent delivery vehicle for therapeutics to inflammatory diseases.[54] It is likely that DCN also accumulated in the skin due to highly activated vasculature, a result of hemorrhagic erosions and active angiogenesis in the RDEB skin.[55] Furthermore, the recombinant DCN has a naturally occurring GAG side chain that, in turn, can bind to α2β1-integrins on angiogenic endothelial cells.[56] Importantly, C7 is a natural ligand of α2β1 integrin.[55] In the absence of C7 in the RDEB skin, α2β1 integrin could be readily available for DCN GAG chain binding and could further enhance the homing of DCN in RDEB skin. However, despite the detection of DCN in RDEB skin lysates, the different accumulation of DCN and DCN-tCRK in the skin suggests distinct tissue penetration mechanisms of these two proteins in the skin. This most probably contributes to their different therapeutic efficacies and highlights the importance of adding cell-penetrating protein domain even for the molecules such as DCN that have intrinsic ability to home to the target organ.[53] Furthermore, tissue penetration and accumulation in the skin offered by NRP-1 could have exposed some of the DCN-tCRK ultimately undetectable in DCN ELISA. Namely, cleaved DCN is found in many skin disorders, and many proteases are known to cleave DCN.[57,58] Among these is granzyme B, which is highly active in human epidermolysis bullosa.[59]

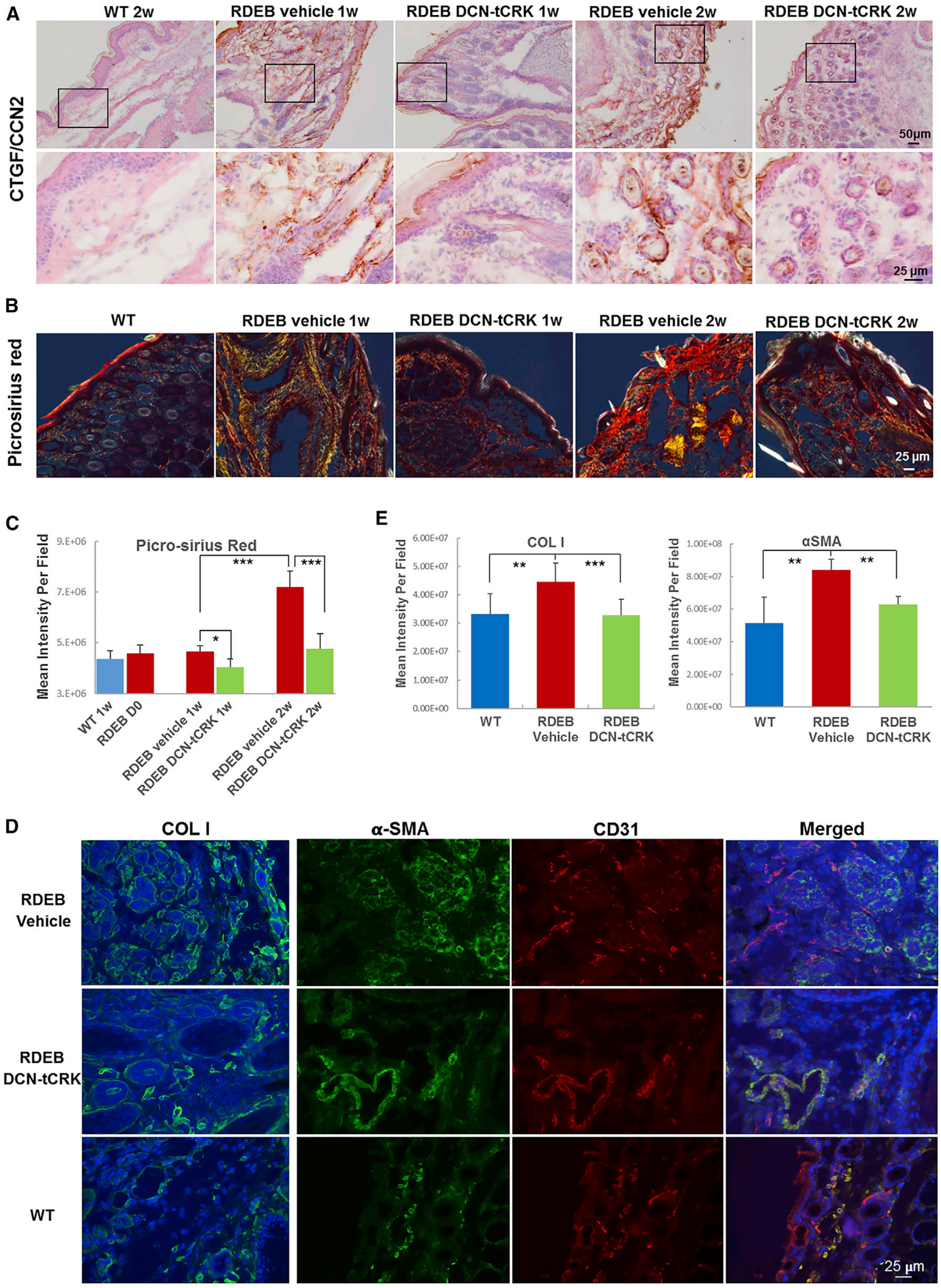

(legend on next page)

(legend on next page)

In summary, we demonstrate that exposure of a cryptic CendR sequence renders novel features in a wound-targeting peptide to home to normal skin in addition to the wounded skin and also provides dermal tissue penetration. This suggests the potential for this peptide (tCRK) to serve as a vehicle for delivering therapeutic molecules in the treatment of systemic dermal diseases. As a proof-ofprinciple study, we demonstrated skin-selective targeting of DCN-tCRK and anti-fibrotic effect of this therapeutic fusion protein in a murine model of RDEB.

MATERIALS AND METHODS

Mice

BALB/cJRj mice (Janvier Labs, Le-Genest-Saint-Isle, France) were used in phage screening, nanoparticle (IONW) homing, and pharmacokinetics. The mice were fed with standard laboratory pellets and water *ad libitum*.

The *col7a1*$^{-/-}$ RDEB mice were used to study the skin-homing and therapeutic function of DCN-tCRK. The *col7a1*$^{-/-}$ RDEB mice were generated by breeding C57BL6/J *col7a1*$^{+/-}$ mice with the genotype determined by polymerase chain reaction (PCR).[60] C57BL6/J *col7a1*$^{+/-}$ mice, kindly provided by Dr. Jouni Uitto at Thomas Jefferson University, were developed by targeted ablation of the *col7a1* gene through out-of-frame deletion.[60]

Wound Healing Models

Eight-week-old male mice (BALB/cJRj), weighing 23–25 g, were used in pharmacokinetics and wound and skin-homing studies. Mice were anesthetized with 4% isoflurane and 0.2 L/min of 100% oxygen mixed with 0.4 L/min air, and the anesthesia was maintained at 2% isoflurane at 0.2 L/min of 100% oxygen mixed with 0.4 L/min air. For the generation of skin wounds, skin was shaved, cleaned, and disinfected. Homing studies were conducted on mice that had circular, 6-mm-diameter, full-thickness excision wounds (including panniculus carnosus muscle) in the dorsal skin.[25] The wounds were first marked by a biopsy punch and then cut with scissors. All skin wounds were left uncovered without a dressing. For the excision wound splinting model, donut-shaped 12-mm silicone splints (Grace Bio-Labs, Bend, OR, USA) were placed around the wounds and affixed with sutures in order to prevent wound contraction.[61,62] The splint excision wounds and the animal welfare were checked every day, and the silicone splints were re-sutured and/or analgesic was administered when needed. The nanoparticle (IONW) synthesis, targeting, and detection are described in Supplemental Materials and Methods.

In Vivo Phage Screening

Phage-homing studies were performed as previously described.[63] Briefly, 8- to 10-week-old mice were injected with a phage clone (1.0×10^9 T7 phage particles [Novagen, Madison, WI, USA] in 100 μL of M9LB medium) through the tail vein and perfused 12 min later through the heart with 1% BSA in Dulbecco's modified Eagle's medium (DMEM, total perfusion volume 75 mL) to remove unbound intravascular phage. Tissue samples were collected and disrupted by a Precellys 24 homogenizer (Bertin Technologies, Montigny-le-Bretoneux, France) using CKMix tubes for wound and skin samples and CK14 tubes for other samples. The cell and tissue suspension was washed in a large volume of 1% BSA-DMEM and centrifuged. For the rescue of the phage particles from tissue samples, cell pellets were lysed with 1% Nonidet P-40 (NP-40) on ice for 5 min and overnight BL21 (415-1b) cultures were added to the lysed cells. The phage particles were rescued by shaking at 37°C for 5 min, after which the samples were titrated using agar plates as previously described.[63]

Recombinant Protein Production

The constructs in the pEFIRES-P expression vector were transfected via lipofection (FuGene 6, Promega, Madison, WI, USA) into HEK293F cells. Positive clones were selected in the culture medium composed of high-glucose DMEM (4.5 g/L) + 2 mM L-alanyl-L-glutamine, 100 IU/mL penicillin (all from Sigma-Aldrich, St. Louis, MO, USA), and 10% FBS (Gibco, Grand Island, NY, USA), in the presence of 5–160 μg/mL puromycin (HyClone, Thermo Fisher Scientific). Established cell lines were maintained in the culture containing 10 μg/mL puromycin.

The validated cells were then resuspended in serum-free OptiCHO medium (Gibco) supplemented with 2 mM L-alanyl-L-glutamine (Sigma) and cultured in square-shaped glass bottles mounted on a rotating shaker at 37°C in a 5% CO_2 atmosphere. After the cells reached a density of $1–2 \times 10^6$ cells/mL, they were cultured further for 4 days at 33°C for recombinant protein expression and secretion to the culture media. The protein was purified from the culture media via the two-step HisTrap purification protocol on the ÄKTA start chromatography system (GE Healthcare, Munich, Germany). A detailed description of the purification and biophysical analysis is in Supplemental Materials and Methods.

Administration of DCN-tCRK and DCN in *col7a1*$^{-/-}$ Mice

The pregnant *col7a1*$^{+/-}$ mice were housed individually and monitored daily before delivery. As intravenous injection in neonatal

Figure 6. DCN-tCRK Administration Suppressed the Development of Fibrosis in *col7a1*$^{-/-}$ Mice

(A) Representative immunohistochemical staining of CTGF in WT and *col7a1*$^{-/-}$ mice at 1 and 2 weeks of age with and without DCN-tCRK treatment. Scale bars, 50 μm (upper panel) and 25 μm (lower panel). (B) Picrosirius red staining of the paw skin from the WT and *col7a1*$^{-/-}$ mice at 1 and 2 weeks of age with and without DCN-tCRK treatment. Picrosirius red images were acquired using polarized light. Scale bar, 25 μm. (C) Quantification of the picrosirius red mean intensity per field acquired with a ×20 objective. Eight or more fields were acquired per section and at least four sections were analyzed per biopsy. Error bars represent SEM. *$p < 0.05$, **$p < 0.01$. (D) Left: representative images of collagen type I (COLI) expression in RDEB and WT skin at 2 weeks. Right: double immunofluorescence staining of α-smooth muscle actin (αSMA, green) and blood vessels (CD31, red) in WT and *col7a1*$^{-/-}$ mice at 2 weeks of age with and without DCN-tCRK treatment. Nuclei were counterstained by DAPI. Scale bar, 25 μm. (E) Quantification of mean immunostaining intensity for COLI and αSMA expression on skin sections (n = 3 in each treatment group). *$p \leq 0.05$, **$p < 0.01$, ***$p < 0.001$.

mice is technically challenging and often yields inconsistent results, we chose to inject within 24 h of birth the first dose of DCN-tCRK and DCN (5 μg in 15 μL of PBS, corresponding to ~5 mg/kg) into the liver of the *col7a1*$^{-/-}$ mice, since liver is a primary site of hematopoiesis in fetal and neonatal mice, and the human cells have been shown to rapidly enter the circulation after intrahepatic injection.[30,31] This first dose was followed by repeated i.p. administration of the protein every other day until the mice reached 14 days of age (maximum of seven doses) and the dose was increased to 10 μg when the mice became a week old. The mice were monitored every day. All of the experimental *col7a1*$^{-/-}$ mice were genotyped at the time of sample collection.

RT2 Profiler PCR Wound Healing Pathway Analysis

The expressions of genes involved in the mouse wound healing pathway were studied using RT2 Profiler PCR array (QIAGEN, Hilden, Germany). The RT2 Profiler array contains primers for 84 wound-healing genes and 5 housekeeping genes with genomic DNA, reverse-transcriptional, and PCR positive controls in 96-well plates. Total RNA was isolated from whole front paw of WT, RDEB, and DCN- or DCN-tCRK-injected *col7a1*$^{-/-}$ mice (three mice in each group) at day 7. Quality and concentration of RNA were determined with a NanoDrop 200C (Thermo Scientific, Waltham, MA, USA). RNA was treated with genomic DNA elimination mix (QIAGEN). 500 ng of total RNA of each sample was applied for reverse transcription using an RT2 First Strand kit (QIAGEN). cDNA synthesis reaction was combined with 2× RT2 SYBR Green master mix, and 25 μL of this cocktail was dispensed in each well of a 96-well plate. qPCR was run on QuantStudio 5 real-time PCR instrument (Applied Biosystems, Foster City, CA, USA). CT values were exported to an Excel file. The resulting raw data were analyzed using the PCR Array Data Analysis template in the GeneGlobe Data Analysis Center (https://geneglobe.qiagen.com). A gene expression was calculated using the $\Delta\Delta C_T$ method. A fold-change gene expression threshold of 1.5 and a p value threshold of 0.05 were used to analyzed data between WT pups and untreated/treated pups.

Histological and Immunohistochemical Staining and hDCN Quantitation in *col7a1*$^{-/-}$ Mice

Dorsal skin and paws (front and rear) were excised from selected mice, embedded in Tissue-Tec OCT compound (Sakura Finetek, Torrance, CA, USA), and stored at −80°C. 6-μm serial sections were cut for each specimen. Picrosirius red staining and anti-CTGF/CCN2 (connective tissue growth factor) (#ab6992, Abcam, Cambridge, UK) immunohistochemical staining were performed at the Core Histology Lab of New York Medical College. For immunochemical staining of his tag, the sections were fixed in 4% paraformaldehyde and blocked with M.O.M. blocking reagent (Vector Laboratories, Burlingame, CA, USA) (for antibodies raised in mouse) or 10% horse serum (Gibco, Grand Island, NY, USA) with 0.1% Triton X-100 (Sigma, St. Louis, MO, USA). The slides were then incubated with respective primary antibodies, including anti-Col1A (#R1038, Acris, Rockville, MD, USA), anti-αSMA (#14968, Cell Signaling Technology, Danvers, MA, USA), anti-6×-His tag (#R930-25, Thermo Fisher Scientific, Carlsbad, CA, USA), and anti-NRP-1 (#AF566-SP, R&D Systems, Minneapolis, MN, USA), followed by corresponding Alexa Fluor 488 secondary antibodies (Invitrogen, Carlsbad, CA, USA). The slides were then mounted in Vectashield mounting medium containing DAPI (Vector Laboratories, Burlingame, CA, USA). Images were acquired using Nikon 90i Eclipse microscope (Nikon Instruments, NY, USA) using the same settings between the different groups in each set of experiments. Intensity of the immunostaining per field was measured using NIS-Elements AR software, following the user's guide. The RGB images were used for the quantitation of picrosirius red staining, and the threshold was defined by choosing reference points within the image.

The homing of DCN-tCRK and DCN to skin in *col7a1*$^{-/-}$ mice was determined using a hDCN DuoSet ELISA kit (#DY143, R&D Systems, Minneapolis, MN, USA), according to the manufacturer's recommendations. Tissue biopsies were snap-frozen in liquid nitrogen, ground with a precooled pestle, and homogenized with lysis buffer (1% Tween 20, protease inhibitor cocktail, DNase, and RNase in PBS). After centrifugation at 12,000 × *g* for 10 min at 4°C, the supernatant was collected and quantitated for total protein concentration with the Bio-Rad DC protein assay (Bio-Rad, Hercules, CA, USA). Sera from *col7a1*$^{-/-}$ mice with and without DCN-tCRK or DCN administration were diluted 1:20 in sample diluent before applying to the assay.

Statistical Analysis

Kaplan-Meier analysis was applied to determine the median lifespan, and a log rank (Mantel-Cox) test was used to compare survival between different experimental groups (GraphPad Prism 6). A Kruskal-Wallis test and Bonferroni *post hoc* correction for pairwise comparisons were used to study phage homing, and a Student's unpaired t test was used for DCN-tCRK binding to NRP-1. p values less than 0.05 were considered significant. SPSS version 24 was used for these tests.

Study Approval

All animal experiments with the BALB/cJRj mice were performed in accordance with protocols approved by the National Animal Ethics Committee of Finland, and all animal studies with the *col7a1*$^{-/-}$ RDEB were conducted using protocols approved by New York Medical College Institutional Animal Care and Use Committee (IACUC).

Additional Methods

Generation of phage clones, synthesis of nanoparticles (IONWs), *in vitro* binding analyses, nanoparticle targeting studies, cloning of the DCN fusion proteins, recombinant protein purification and biophysical analyses, collagen gel contraction assay, as well as pharmacokinetics were performed using standard, published methods[8,28,64,65] and are described in detail in Supplemental Materials and Methods.

SUPPLEMENTAL INFORMATION

Supplemental Information can be found online at https://doi.org/10.1016/j.ymthe.2020.05.017.

AUTHOR CONTRIBUTIONS

T.P., M.S.C., T.A.H.J., and Y.L. designed the research. L.I., T.P., U.M., S.P., P.L., A.T., A.P., O.R., and A.P. performed the research. L.I., T.P., U.M., P.L., T.J., and Y.L. analyzed the data. T.A.H.J. and Y.L. wrote the manuscript. Y.L., T.P., L.I., U.M., and P.L. generated the figures. All authors reviewed and accepted the text of the manuscript.

CONFLICTS OF INTEREST

The authors declare no competing interests.

ACKNOWLEDGMENTS

We thank Dr. Venkata Ramana Kotamraju for peptide synthesis, and Marianne Karlsberg, Guillermina Garcia, and Marja-Leena Koskinen for practical support and for performing the histochemical work and immunohistological staining. We are grateful to Dr. Jouni Uitto at Jefferson Medical College (Philadelphia, PA, USA) for the *col7a1*$^{+/-}$ mice, and to Dr. Erkki Ruoslahti at the Sanford Burnham Prebys Medical Discovery Institute (La Jolla, CA, USA) for insightful comments. The authors thank the Tampere Facility of Protein Services and the Tampere Mass Spectrometry Facility as well as the Tampere Imaging Facility and BioCenter Finland for their services. We acknowledge the scientific discussion from colleagues at the Pediatric Cancer Research Laboratory at NYMC and thank Erin Morris, Janet Ayello, and Miguel Muniz for assistance in the preparation of this manuscript. This work was funded by the Pediatric Cancer Research Foundation, the Academy of Finland, the Päivikki and Sakari Sohlberg Foundation, the Tampere Tuberculosis Foundation, the Finnish Medical Foundation, the Instrumentarium Science Foundation, the Juhani Aho Foundation for Medical Research, the Emil Aaltonen Foundation, the Pirkanmaa Hospital District Research Foundation, and the Finnish Cultural Foundation. T.T. was supported by the European Regional Development Fund (Project 2014-2020.4.01.15-0012), by European Research Council grant GLIOGUIDE from the European Regional Development Fund, and by the Estonian Research Council (grants PRG230 and EAG79).

REFERENCES

1. Järvinen, T.A.H., Rashid, J., Valmari, T., May, U., and Ahsan, F. (2017). Systemically administered, target-specific therapeutic recombinant proteins and nanoparticles for regenerative medicine. ACS Biomater. Sci. Eng. *3*, 1273–1282.
2. Ruoslahti, E., Bhatia, S.N., and Sailor, M.J. (2010). Targeting of drugs and nanoparticles to tumors. J. Cell Biol. *188*, 759–768.
3. Ruoslahti, E. (2017). Tumor penetrating peptides for improved drug delivery. Adv. Drug Deliv. Rev. *110-111*, 3–12.
4. Ruoslahti, E. (2004). Vascular zip codes in angiogenesis and metastasis. Biochem. Soc. Trans. *32*, 397–402.
5. Ruoslahti, E. (2017). Access granted: iRGD helps silicasome-encased drugs breach the tumor barrier. J. Clin. Invest. *127*, 1622–1624.
6. Teesalu, T., Sugahara, K.N., Kotamraju, V.R., and Ruoslahti, E. (2009). C-end rule peptides mediate neuropilin-1-dependent cell, vascular, and tissue penetration. Proc. Natl. Acad. Sci. USA *106*, 16157–16162.
7. Sugahara, K.N., Teesalu, T., Karmali, P.P., Kotamraju, V.R., Agemy, L., Girard, O.M., Hanahan, D., Mattrey, R.F., and Ruoslahti, E. (2009). Tissue-penetrating delivery of compounds and nanoparticles into tumors. Cancer Cell *16*, 510–520.
8. Sugahara, K.N., Teesalu, T., Karmali, P.P., Kotamraju, V.R., Agemy, L., Greenwald, D.R., and Ruoslahti, E. (2010). Coadministration of a tumor-penetrating peptide enhances the efficacy of cancer drugs. Science *328*, 1031–1035.
9. Hilal, L., Rochat, A., Duquesnoy, P., Blanchet-Bardon, C., Wechsler, J., Martin, N., Christiano, A.M., Barrandon, Y., Uitto, J., Goossens, M., et al. (1993). A homozygous insertion-deletion in the type VII collagen gene (COL7A1) in Hallopeau-Siemens dystrophic epidermolysis bullosa. Nat. Genet. *5*, 287–293.
10. Bruckner-Tuderman, L., McGrath, J.A., Robinson, E.C., and Uitto, J. (2013). Progress in epidermolysis bullosa research: summary of DEBRA International Research Conference 2012. J. Invest. Dermatol. *133*, 2121–2126.
11. Kiuru, M., Itoh, M., Cairo, M.S., and Christiano, A.M. (2010). Bone marrow stem cell therapy for recessive dystrophic epidermolysis bullosa. Dermatol. Clin. *28*, 371–382, xii–xiii.
12. Nyström, A., Bernasconi, R., and Bornert, O. (2018). Therapies for genetic extracellular matrix diseases of the skin. Matrix Biol. *71-72*, 330–347.
13. Uitto, J. (2019). Toward treatment and cure of epidermolysis bullosa. Proc. Natl. Acad. Sci. USA *116*, 26147–26149.
14. Rashidghamat, E., and McGrath, J.A. (2017). Novel and emerging therapies in the treatment of recessive dystrophic epidermolysis bullosa. Intractable Rare Dis. Res. *6*, 6–20.
15. Nyström, A., and Bruckner-Tuderman, L. (2018). Injury- and inflammation-driven skin fibrosis: the paradigm of epidermolysis bullosa. Matrix Biol. *68-69*, 547–560.
16. Cianfarani, F., Zambruno, G., Castiglia, D., and Odorisio, T. (2017). Pathomechanisms of altered wound healing in recessive dystrophic epidermolysis bullosa. Am. J. Pathol. *187*, 1445–1453.
17. Knaup, J., Gruber, C., Krammer, B., Ziegler, V., Bauer, J., and Verwanger, T. (2011). TGFβ-signaling in squamous cell carcinoma occurring in recessive dystrophic epidermolysis bullosa. Anal. Cell. Pathol. (Amst.) *34*, 339–353.
18. Odorisio, T., Di Salvio, M., Orecchia, A., Di Zenzo, G., Piccinni, E., Cianfarani, F., Travaglione, A., Uva, P., Bellei, B., Conti, A., et al. (2014). Monozygotic twins discordant for recessive dystrophic epidermolysis bullosa phenotype highlight the role of TGF-β signalling in modifying disease severity. Hum. Mol. Genet. *23*, 3907–3922.
19. Nyström, A., Thriene, K., Mittapalli, V., Kern, J.S., Kiritsi, D., Dengjel, J., and Bruckner-Tuderman, L. (2015). Losartan ameliorates dystrophic epidermolysis bullosa and uncovers new disease mechanisms. EMBO Mol. Med. *7*, 1211–1228.
20. Liao, Y., Ivanova, L., Zhu, H., Plumer, T., Hamby, C., Mehta, B., Gevertz, A., Christiano, A.M., McGrath, J.A., and Cairo, M.S. (2018). Cord blood-derived stem cells suppress fibrosis and may prevent malignant progression in recessive dystrophic epidermolysis bullosa. Stem Cells *36*, 1839–1850.
21. Reed, C.C., and Iozzo, R.V. (2002). The role of decorin in collagen fibrillogenesis and skin homeostasis. Glycoconj. J. *19*, 249–255.
22. Järvinen, T.A., and Prince, S. (2015). Decorin: a growth factor antagonist for tumor growth inhibition. BioMed Res. Int. *2015*, 654765.
23. Järvinen, T.A.H., and Ruoslahti, E. (2019). Generation of a multi-functional, target organ-specific, anti-fibrotic molecule by molecular engineering of the extracellular matrix protein, decorin. Br. J. Pharmacol. *176*, 16–25.
24. Cianfarani, F., De Domenico, E., Nyström, A., Mastroeni, S., Abeni, D., Baldini, E., Ulisse, S., Uva, P., Bruckner-Tuderman, L., Zambruno, G., et al. (2019). Decorin counteracts disease progression in mice with recessive dystrophic epidermolysis bullosa. Matrix Biol. *81*, 3–16.
25. Järvinen, T.A., and Ruoslahti, E. (2007). Molecular changes in the vasculature of injured tissues. Am. J. Pathol. *171*, 702–711.
26. Agemy, L., Sugahara, K.N., Kotamraju, V.R., Gujraty, K., Girard, O.M., Kono, Y., Mattrey, R.F., Park, J.H., Sailor, M.J., Jimenez, A.I., et al. (2010). Nanoparticle-induced vascular blockade in human prostate cancer. Blood *116*, 2847–2856.
27. Park, J.H., von Maltzahn, G., Ruoslahti, E., Bhatia, S.N., and Sailor, M.J. (2008). Micellar hybrid nanoparticles for simultaneous magnetofluorescent imaging and drug delivery. Angew. Chem. Int. Ed. Engl. *47*, 7284–7288.
28. Park, J.H., von Maltzahn, G., Zhang, L., Schwartz, M.P., Ruoslahti, E., Bhatia, S.N., and Sailor, M.J. (2008). Magnetic iron oxide nanoworms for tumor targeting and imaging. Adv. Mater. *20*, 1630–1635.

29. Scott, P.G., Grossmann, J.G., Dodd, C.M., Sheehan, J.K., and Bishop, P.N. (2003). Light and X-ray scattering show decorin to be a dimer in solution. J. Biol. Chem. *278*, 18353–18359.

30. Liao, Y., Ivanova, L., Zhu, H., Yahr, A., Ayello, J., van de Ven, C., Rashad, A., Uitto, J., Christiano, A.M., and Cairo, M.S. (2015). Rescue of the mucocutaneous manifestations by human cord blood derived nonhematopoietic stem cells in a mouse model of recessive dystrophic epidermolysis bullosa. Stem Cells *33*, 1807–1817.

31. Liao, Y., Ivanova, L., Sivalenka, R., Plumer, T., Zhu, H., Zhang, X., Christiano, A.M., McGrath, J.A., Gurney, J.P., and Cairo, M.S. (2018). Efficacy of human placental-derived stem cells in collagen vii knockout (recessive dystrophic epidermolysis bullosa) animal model. Stem Cells Transl. Med. *7*, 530–542.

32. Järvinen, T.A., and Ruoslahti, E. (2010). Target-seeking antifibrotic compound enhances wound healing and suppresses scar formation in mice. Proc. Natl. Acad. Sci. USA *107*, 21671–21676.

33. Roth, L., Agemy, L., Kotamraju, V.R., Braun, G., Teesalu, T., Sugahara, K.N., Hamzah, J., and Ruoslahti, E. (2012). Transtumoral targeting enabled by a novel neuropilin-binding peptide. Oncogene *31*, 3754–3763.

34. Fukushima, D., Bützow, R., Hildebrand, A., and Ruoslahti, E. (1993). Localization of transforming growth factor β binding site in betaglycan. Comparison with small extracellular matrix proteoglycans. J. Biol. Chem. *268*, 22710–22715.

35. Hildebrand, A., Romarís, M., Rasmussen, L.M., Heinegård, D., Twardzik, D.R., Border, W.A., and Ruoslahti, E. (1994). Interaction of the small interstitial proteoglycans biglycan, decorin and fibromodulin with transforming growth factor β. Biochem. J. *302*, 527–534.

36. Vial, C., Gutiérrez, J., Santander, C., Cabrera, D., and Brandan, E. (2011). Decorin interacts with connective tissue growth factor (CTGF)/CCN2 by LRR12 inhibiting its biological activity. J. Biol. Chem. *286*, 24242–24252.

37. Daniels, J.T., Schultz, G.S., Blalock, T.D., Garrett, Q., Grotendorst, G.R., Dean, N.M., and Khaw, P.T. (2003). Mediation of transforming growth factor-β_1-stimulated matrix contraction by fibroblasts: a role for connective tissue growth factor in contractile scarring. Am. J. Pathol. *163*, 2043–2052.

38. Border, W.A., Noble, N.A., Yamamoto, T., Harper, J.R., Yamaguchi, Y.u., Pierschbacher, M.D., and Ruoslahti, E. (1992). Natural inhibitor of transforming growth factor-β protects against scarring in experimental kidney disease. Nature *360*, 361–364.

39. Border, W.A., and Ruoslahti, E. (1992). Transforming growth factor-β in disease: the dark side of tissue repair. J. Clin. Invest. *90*, 1–7.

40. Abdullatif, A.M., Macky, T.A., Abdullatif, M.M., Nassar, K., Grisanti, S., Mortada, H.A., and Soliman, M.M. (2018). Intravitreal decorin preventing proliferative vitreoretinopathy in perforating injuries: a pilot study. Graefes Arch. Clin. Exp. Ophthalmol. *256*, 2473–2481.

41. Danielson, K.G., Baribault, H., Holmes, D.F., Graham, H., Kadler, K.E., and Iozzo, R.V. (1997). Targeted disruption of decorin leads to abnormal collagen fibril morphology and skin fragility. J. Cell Biol. *136*, 729–743.

42. Gubbiotti, M.A., Vallet, S.D., Ricard-Blum, S., and Iozzo, R.V. (2016). Decorin interacting network: a comprehensive analysis of decorin-binding partners and their versatile functions. Matrix Biol. *55*, 7–21.

43. Nareyeck, G., Seidler, D.G., Troyer, D., Rauterberg, J., Kresse, H., and Schönherr, E. (2004). Differential interactions of decorin and decorin mutants with type I and type VI collagens. Eur. J. Biochem. *271*, 3389–3398.

44. Andrianifahanana, M., Wilkes, M.C., Gupta, S.K., Rahimi, R.A., Repellin, C.E., Edens, M., Wittenberger, J., Yin, X., Maidl, E., Becker, J., and Leof, E.B. (2013). Profibrotic TGFβ responses require the cooperative action of PDGF and ErbB receptor tyrosine kinases. FASEB J. *27*, 4444–4454.

45. Lam, A.P., and Gottardi, C.J. (2011). β-Catenin signaling: a novel mediator of fibrosis and potential therapeutic target. Curr. Opin. Rheumatol. *23*, 562–567.

46. Grotendorst, G.R., Rahmanie, H., and Duncan, M.R. (2004). Combinatorial signaling pathways determine fibroblast proliferation and myofibroblast differentiation. FASEB J. *18*, 469–479.

47. Grotendorst, G.R., and Duncan, M.R. (2005). Individual domains of connective tissue growth factor regulate fibroblast proliferation and myofibroblast differentiation. FASEB J. *19*, 729–738.

48. Neill, T., Schaefer, L., and Iozzo, R.V. (2012). Decorin: a guardian from the matrix. Am. J. Pathol. *181*, 380–387.

49. Buraschi, S., Pal, N., Tyler-Rubinstein, N., Owens, R.T., Neill, T., and Iozzo, R.V. (2010). Decorin antagonizes Met receptor activity and down-regulates β-catenin and Myc levels. J. Biol. Chem. *285*, 42075–42085.

50. Seidler, D.G., Goldoni, S., Agnew, C., Cardi, C., Thakur, M.L., Owens, R.T., McQuillan, D.J., and Iozzo, R.V. (2006). Decorin protein core inhibits in vivo cancer growth and metabolism by hindering epidermal growth factor receptor function and triggering apoptosis via caspase-3 activation. J. Biol. Chem. *281*, 26408–26418.

51. Goldoni, S., Seidler, D.G., Heath, J., Fassan, M., Baffa, R., Thakur, M.L., Owens, R.T., McQuillan, D.J., and Iozzo, R.V. (2008). An antimetastatic role for decorin in breast cancer. Am. J. Pathol. *173*, 844–855.

52. Ma, W., Cai, S., Du, J., Tan, Y., Chen, H., Guo, Z., Hu, H., Fang, R., and Cai, S. (2008). SDF-1/54-DCN: a novel recombinant chimera with dual inhibitory effects on proliferation and chemotaxis of tumor cells. Biol. Pharm. Bull. *31*, 1086–1091.

53. Järvinen, T.A., and Pemmari, T. (2020). Systemically administered, target-specific, multi-functional therapeutic recombinant proteins in regenerative medicine. Nanomaterials *10*, 226, https://doi.org/10.3390/nano10020226.

54. Katsumata, K., Ishihara, J., Mansurov, A., Ishihara, A., Raczy, M.M., Yuba, E., and Hubbell, J.A. (2019). Targeting inflammatory sites through collagen affinity enhances the therapeutic efficacy of anti-inflammatory antibodies. Sci. Adv *5*, eaay1971.

55. Martins, V.L., Caley, M.P., Moore, K., Szentpetery, Z., Marsh, S.T., Murrell, D.F., Kim, M.H., Avari, M., McGrath, J.A., Cerio, R., et al. (2015). Suppression of TGFβ and angiogenesis by type VII collagen in cutaneous SCC. J. Natl. Cancer Inst. *108*, djv293.

56. Fiedler, L.R., Schönherr, E., Waddington, R., Niland, S., Seidler, D.G., Aeschlimann, D., and Eble, J.A. (2008). Decorin regulates endothelial cell motility on collagen I through activation of insulin-like growth factor I receptor and modulation of α2β1 integrin activity. J. Biol. Chem. *283*, 17406–17415.

57. Carrino, D.A., Onnerfjord, P., Sandy, J.D., Cs-Szabo, G., Scott, P.G., Sorrell, J.M., Heinegård, D., and Caplan, A.I. (2003). Age-related changes in the proteoglycans of human skin. Specific cleavage of decorin to yield a major catabolic fragment in adult skin. J. Biol. Chem. *278*, 17566–17572.

58. Turner, C.T., Lim, D., and Granville, D.J. (2019). Granzyme B in skin inflammation and disease. Matrix Biol. *75–76*, 126–140.

59. Russo, V., Klein, T., Lim, D.J., Solis, N., Machado, Y., Hiroyasu, S., Nabai, L., Shen, Y., Zeglinski, M.R., Zhao, H., et al. (2018). Granzyme B is elevated in autoimmune blistering diseases and cleaves key anchoring proteins of the dermal-epidermal junction. Sci. Rep. *8*, 9690.

60. Heinonen, S., Männikkö, M., Klement, J.F., Whitaker-Menezes, D., Murphy, G.F., and Uitto, J. (1999). Targeted inactivation of the type VII collagen gene (Col7a1) in mice results in severe blistering phenotype: a model for recessive dystrophic epidermolysis bullosa. J. Cell Sci. *112*, 3641–3648.

61. Barker, H., Aaltonen, M., Pan, P., Vähätupa, M., Kaipiainen, P., May, U., Prince, S., Uusitalo-Järvinen, H., Waheed, A., Pastoreková, S., et al. (2017). Role of carbonic anhydrases in skin wound healing. Exp. Mol. Med. *49*, e334.

62. Ketomäki, T., Vähätupa, M., May, U., Pemmari, T., Ruikka, E., Hietamo, J., Kaipiainen, P., Barker, H., Parkkila, S., Uusitalo-Järvinen, H., and Järvinen, T.A.H. (2019). R-Ras regulates vascular permeability, but not overall healing in skin wounds. Exp. Dermatol. *28*, 202–206.

63. Järvinen, T.A. (2012). Design of target-seeking antifibrotic compounds. Methods Enzymol. *509*, 243–261.

64. Urakami, T., Järvinen, T.A., Toba, M., Sawada, J., Ambalavanan, N., Mann, D., McMurtry, I., Oka, M., Ruoslahti, E., and Komatsu, M. (2011). Peptide-directed highly selective targeting of pulmonary arterial hypertension. Am. J. Pathol. *178*, 2489–2495.

65. Toba, M., Alzoubi, A., O'Neill, K., Abe, K., Urakami, T., Komatsu, M., Alvarez, D., Järvinen, T.A., Mann, D., Ruoslahti, E., et al. (2014). A novel vascular homing peptide strategy to selectively enhance pulmonary drug efficacy in pulmonary arterial hypertension. Am. J. Pathol. *184*, 369–375.

YMTHE, Volume *28*

Supplemental Information

Exposed CendR Domain in Homing Peptide Yields Skin-Targeted Therapeutic in Epidermolysis Bullosa

Toini Pemmari, Larisa Ivanova, Ulrike May, Prakash Lingasamy, Allan Tobi, Anja Pasternack, Stuart Prince, Olli Ritvos, Shreya Makkapati, Tambet Teesalu, Mitchell S. Cairo, Tero A.H. Järvinen, and Yanling Liao

Supplemental Figures

Figure S1

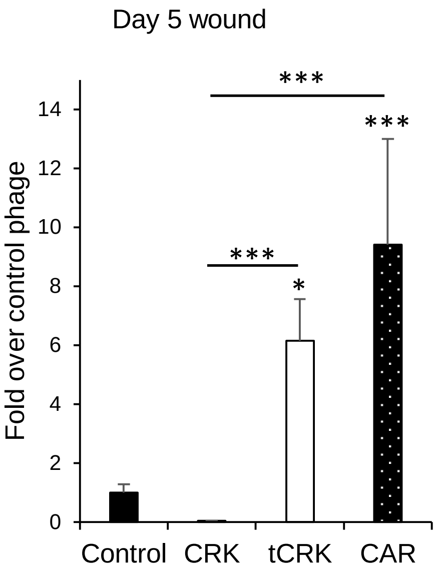

Figure S1: Homing of tCRK phage in 5 days old wound. The amounts are represented as fold over control phage. Error bars represent SEM, *p < 0.05, **p < 0.01, ***p < 0.001, Kruskal-Wallis test with Bonferroni post hoc, n = 14 for tCRK and CAR, n = 5 for CRK, and n = 11 for control phage.

Figure S2

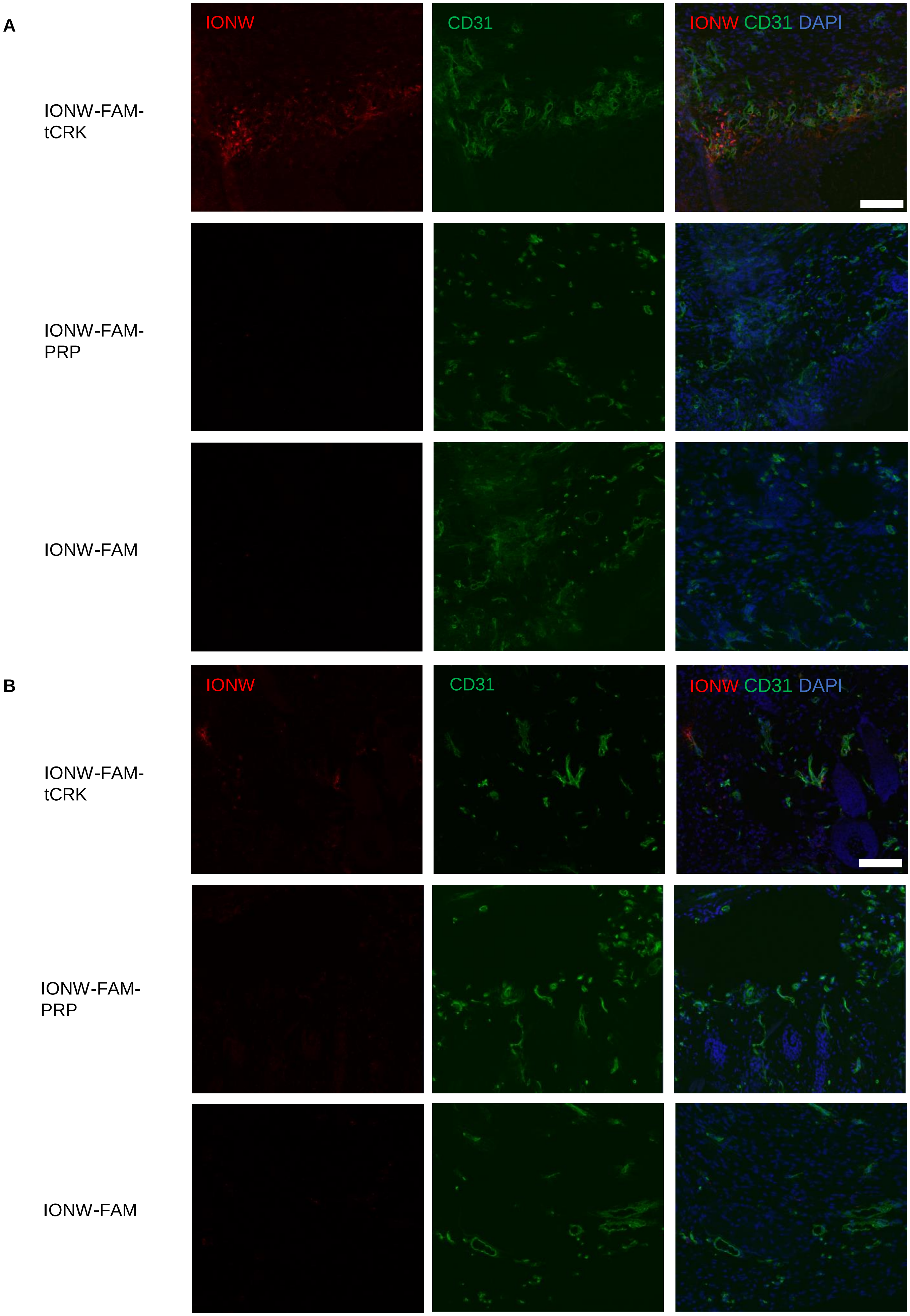
A
IONW
CD31
IONW CD31 DAPI
IONW-FAM-tCRK
IONW-FAM-PRP
IONW-FAM
B
IONW
CD31
IONW CD31 DAPI
IONW-FAM-tCRK
IONW-FAM-PRP
IONW-FAM

Figure S2: Homing of tCRK coated nanoparticles to skin wounds. Representative images of immunohistochemical staining of excision wound (A) and excision wound with splints (B) samples from Balb/c mice with anti-FITC (red) to detect the *i.v.* injected FAM-labeled IONWs coated with tCRK (12 mg/kg), a control peptide PRP (16 mg/kg) or no peptide (FAM alone, 13 mg/kg). The localization of blood vessels is depicted by anti-CD31 (green) and nuclei are stained with DAPI (blue). Scale bar 100 µm. Representative images from three independent experiments.

Figure S3

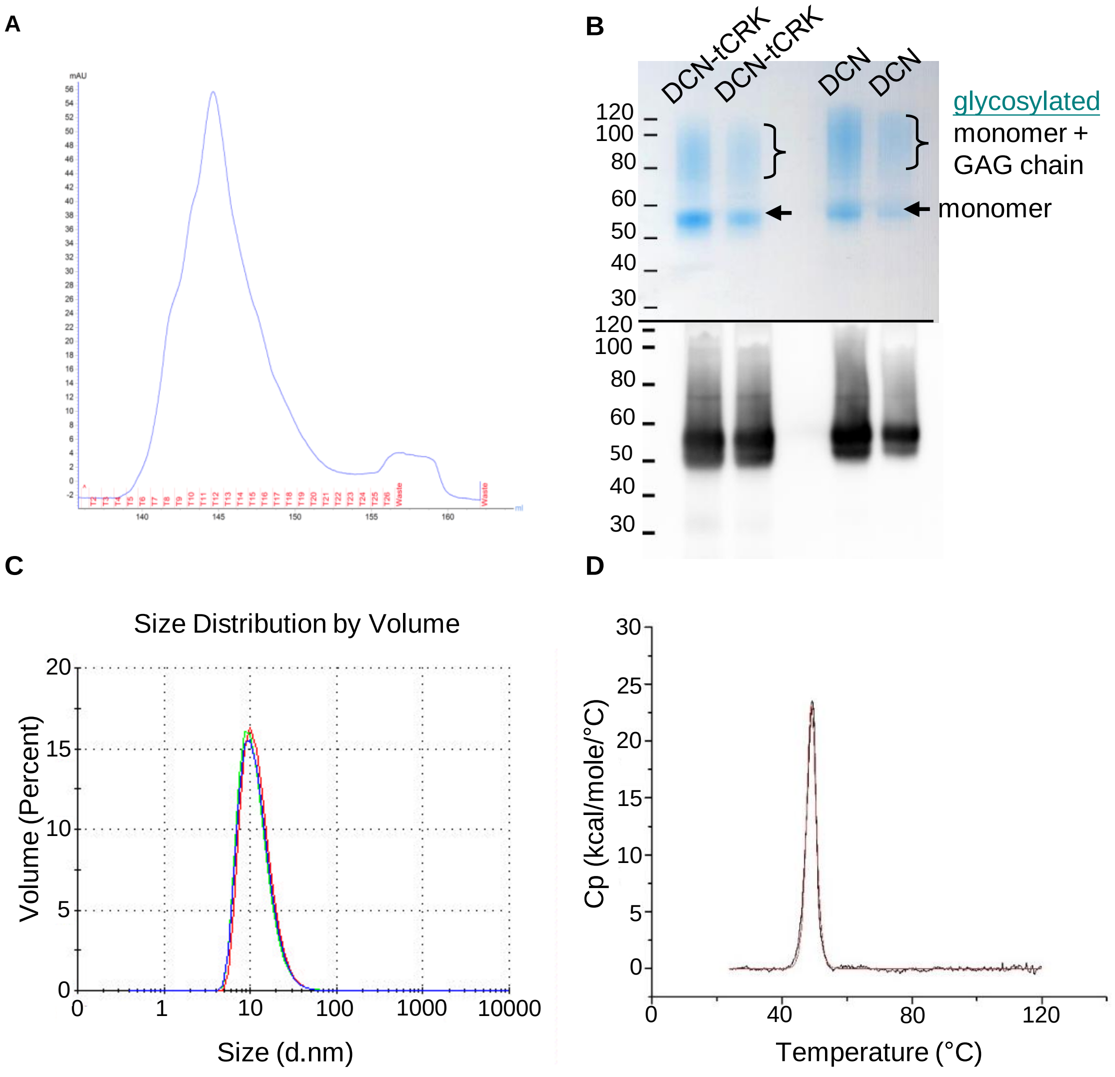

Figure S3: Recombinant protein production and characterization of DCN-tCRK. (A) An example of a purification chromatogram after the HisTrap HP column step on the Äkta Start shows one big peak, of which all peak fractions were used for further processing. (B) Coomassie-stained reduced SDS-Page gel (upper panel) and Western blot (lower panel) of purified DCN-tCRK are shown alongside the already published/characterized DCN. On the SDS gel 2 and 1 µg of protein were loaded; for Western blot analysis 1 and 0.5 µg of protein were applied. Monomeric forms of the proteins, as well as forms including the GAG side chains are visible. (C) Dynamic light scattering (DLS) measurements (n = 3) on the hydrodynamic diameter of DCN-tCRK. (D) Differential scanning calorimetry (DSC) curve for the melting temperature of DCN-tCRK.

Figure S4

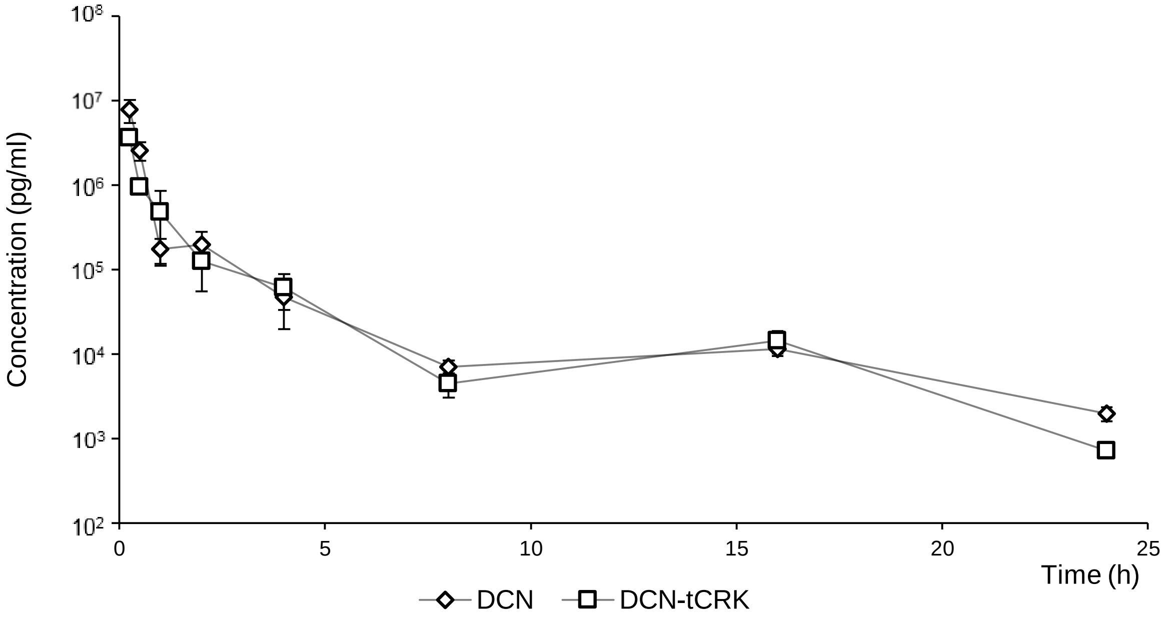

Figure S4: The pharmacokinetics of DCN-tCRK and DCN. 5 mg/kg of DCN-tCRK or DCN was injected *i.v.* Blood samples were gathered and analyzed with standard ELISA for human DCN from eight time points. Error bars represent SD, n = 4 per group.

Figure S5

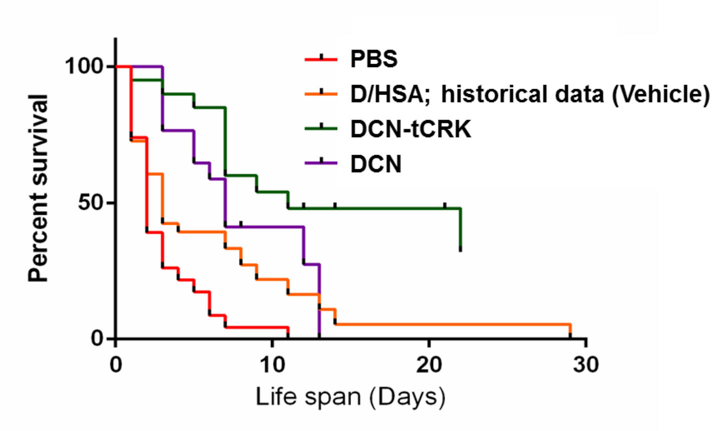

Figure S5: Kaplan-Meier survival analysis of the *col7a1*$^{-/-}$ mice comparing the historical survival after dextran/human serum albumin (D/HSA; median life span: 3 days; n = 29, orange line; historical data[1]) administration with the survival after DCN-tCRK (median life span: 11 days; n = 21, green line) and DCN (median life span: 7 days; n = 17, purple line) and PBS (median life span: 2 days; n = 24, red line) administration.

Figure S6

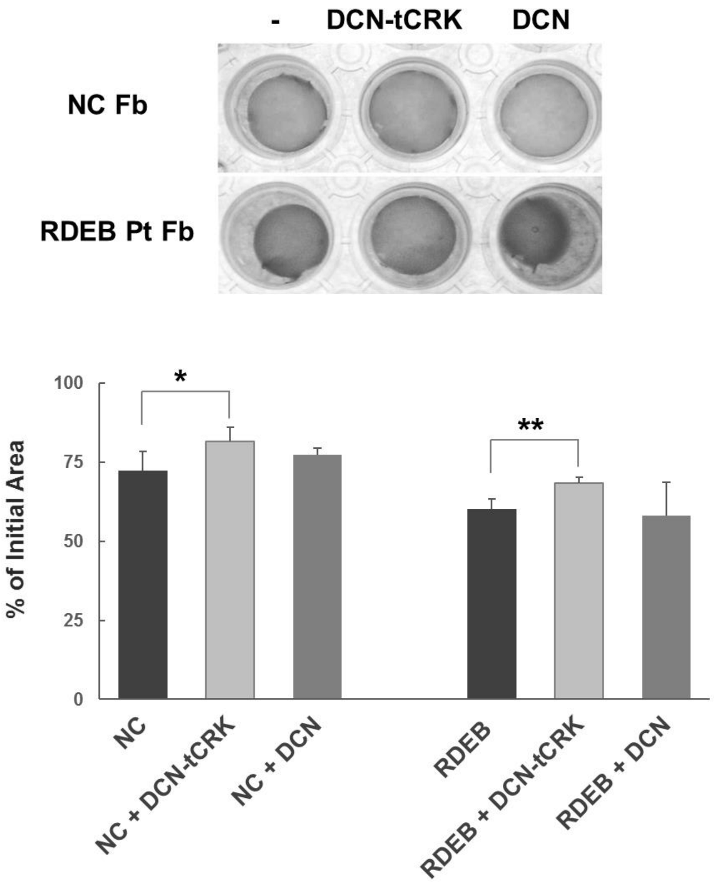

Figure S6: Collagen lattice contraction assay. Upper, representative images of human normal fibroblasts and RDEB patient-derived fibroblasts 48 hours after seeding in collagen gels, with and without addition of DCN and DCN-tCRK at a final concentration of 75 µM. Bottom, contraction of collagen gels, calculated as percentage of contraction compared with the initial area. Data (n = 3) are presented as mean ± SEM. * $p < 0.05$, ** $p < 0.001$.

Supplemental Tables

Table S1: The sequence of human DCN and the tCRK sequence in the C-terminus analyzed by mass spectrometry. The green letters indicate the peptides that were found to be specific to human DCN and the blue letters indicate the specific C-end terminus including the tCRK sequence, which is further indicated with bold.

N	Unused	Total	% Cov	Accession #	Name	Peptides (95%)
1	54,45	54,45	45,7	sp|P07585|PGS2_HUMAN	Decorin OS=Homo sapiens GN=DCN PE=1 SV=1	48
					MKATIILLLLAQVSWAGPFQQRGLFDFMLEDEAS<br>GIGPEVPDDRDFEPSLGPVCPFRCQCHLRVVQCSD<br>LGLDKVPKDLPPDTTLLDLQNNKITEIKDGDFKNL<br>KNLHALILVNNKISKVSPGAFTPLVKLERLYLSKN<br>QLKELPEKMPKTLQELRAHENEITKVRKVTFNGL<br>NQMIVIELGTNPLKSSGIENGAFQGMKKLSYIRIA<br>DTNITSIPQGLPPSLTELHLDGNKISRVDAASLKGL<br>NNLAKLGLSFNSISAVDNGSLANTPHLRELHLDN<br>NKLTRVPGGLAEHKYIQVVYLHNNNISVVGSSDF<br>CPPGHNTKKASYSGVSLFSNPVQYWEIQPSTFRCV<br>YVRSAIQLGNYK	
1	66	66	46,7	sp|P07585|PGS2_HUMAN	Decorin OS=Homo sapiens OX=9606 GN=DCN PE=1 SV=1	63
					MKATIILLLLAQVSWAGPFQQRGLFDFMLEDEAS<br>GIGPEVPDDRDFEPSLGPVCPFRCQCHLRVVQCSD<br>LGLDKVPKDLPPDTTLLDLQNNKITEIKDGDFKNL<br>KNLHALILVNNKISKVSPGAFTPLVKLERLYLSKN<br>QLKELPEKMPKTLQELRAHENEITKVRKVTFNGL<br>NQMIVIELGTNPLKSSGIENGAFQGMKKLSYIRIA<br>DTNITSIPQGLPPSLTELHLDGNKISRVDAASLKGL<br>NNLAKLGLSFNSISAVDNGSLANTPHLRELHLDN<br>NKLTRVPGGLAEHKYIQVVYLHNNNISVVGSSDF<br>CPPGHNTKKASYSGVSLFSNPVQYWEIQPSTFRCV<br>YVRSAIQLGNYKGSEF**CRKDK**	
					GREEN: Peptides that were found to be specific to human DCN; BLUE: specific for the C-terminus including the tCRK sequence (CRKDK)	

Table S2: Fold changes of gene expression in vehicle, DCN and DCN-tCRK treated *col7a*$^{-/-}$ skin relative to the WT and P values. N/A indicates average threshold cycle either not determined or greater than the defined cut-off. The genes that are significantly upregulated as compared to the WT are bolded and the genes that are significantly upregulated only in DCN-treated *col7a*$^{-/-}$ skin were marked as red.

Gene Symbols	Vehicle RDEB vs WT		DCN-tCRK RDEB vs WT		DCN RDEB vs. WT	
	Fold changes	P value	Fold changes	P value	Fold changes	P value
Acta2	1.55	0.695	0.84	0.672	N/A	N/A
Actc1	1.19	0.633	1.36	0.815	0.65	0.426
Angpt1	1.54	0.545	0.72	0.927	2.94	0.271
Ccl12	8.28	0.375	N/A	N/A	N/A	N/A
Ccl7	0.66	0.701	0.74	0.594	**3.07**	**0.001**
Cd40lg	0.85	0.783	N/A	N/A	N/A	N/A
Cdh1	2.58	0.076	1.76	0.439	3.20	0.062
Col14a1	0.89	0.690	0.84	0.737	1.02	0.751
Col1a1	1.79	0.405	1.45	0.396	1.16	0.893
Col1a2	0.82	0.897	0.53	0.830	1.2	0.722
Col3a1	1.06	0.885	0.6	0.74	1.71	0.501
Col4a1	0.94	0.487	0.25	0.356	N/A	N/A
Col4a3	2.96	0.203	1.08	0.655	3.16	0.068
Col5a1	4.47	0.195	1.21	0.582	1.12	0.226
Col5a2	1.24	0.554	0.61	0.795	1.34	0.572
Col5a3	1.86	0.370	1.8	0.410	1.83	0.228
Csf2	0.91	0.849	0.7	0.762	**45.86**	**0.004**
Csf3	1.36	0.439	2.19	0.332	**7.04**	**0.049**
**Ctgf**	**3.26**	**0.037**	2.44	0.281	**4.55**	**0.050**
**Ctnnb1**	**3.08**	**0.009**	2.25	0.349	**3.79**	**0.009**
**Ctsg**	**3.15**	**0.002**	1.77	0.292	**9.23**	**0.021**
Ctsk	1.7	0.266	0.69	0.897	1.4	0.422
Ctsl	2.25	0.285	1.01	0.883	2.42	0.207
Cxcl1	4.97	0.106	2.05	0.326	12.49	0.085
Cxcl11	N/A	N/A	N/A	N/A	N/A	N/A
**Cxcl3**	**3.41**	**0.009**	1.98	0.073	**26.44**	**0.047**
Cxcl5	1.25	0.449	1.35	0.482	**7.03**	**0.023**
Egf	0.66	0.218	0.54	0.816	0.67	0.031
**Egfr**	**4.68**	**0.015**	1.75	0.367	**2.20**	**0.039**
F13a1	1.96	0.493	0.82	0.747	1.78	0.484
F3	3.04	0.114	1.74	0.423	2.99	0.172
Fga	N/A	N/A	N/A	N/A	N/A	N/A
Fgf10	1.84	0.164	1.15	0.543	1.56	0.317
Fgf2	2.2	0.125	1.17	0.633	3.49	0.164
Fgf7	1.23	0.761	1.1	0.730	1.13	0.951
Hbegf	2.69	0.154	1.11	0.658	**4.01**	**0.026**
Hgf	3.36	0.377	0.25	0.259	8.12	0.374
Ifng	N/A	N/A	N/A	N/A	N/A	N/A
Igf1	1.22	0.606	0.71	0.694	1.79	0.337
Il10	2.00	0.408	1.1	0.979	3.26	0.235
Il1b	4.12	0.262	1.1	0.608	100.49	0.132
Il2	N/A	N/A	N/A	N/A	N/A	N/A
**Il4**	**4.29**	**0.015**	2.08	0.302	**13.75**	**0.006**
Il6	1.62	0.358	1.34	0.571	8.75	0.082
Il6st	1.81	0.313	1.58	0.481	2.35	0.141
Itga1	1.58	0.42	0.95	0.909	1.2	0.913
Itga2	2.24	0.16	1.45	0.547	2.17	0.197
Itga3	4.57	0.104	2.17	0.172	**2.01**	**0.003**
Itga4	2.26	0.746	0.447	0.304	N/A	N/A

Itga5	3.37	0.207	1.62	0.397	2.39	0.127
Itga6	2.41	0.106	1.39	0.507	4.31	0.295
Itgav	2.25	0.148	1.32	0.557	2.14	0.159
Itgb1	2.29	0.178	0.69	0.769	**2.90**	**0.048**
**Itgb3**	**1.57**	**0.001**	0.57	0.084	1.24	0.391
**Itgb5**	**5.78**	**0.050**	3.79	0.266	3.29	0.181
Itgb6	1.26	0.228	0.62	0.680	2.68	0.329
Mapk1	2.01	0.167	1.42	0.488	**3.44**	**0.012**
**Mapk3**	**2.18**	**0.040**	1.33	0.504	1.75	0.267
Mif	0.4	0.709	1.64	0.439	1.1	0.986
Mmp1a	1.27	0.451	0.95	0.713	0.26	0.013
Mmp2	N/A	N/A	N/A	N/A	N/A	N/A
Mmp7	N/A	N/A	N/A	N/A	N/A	N/A
Mmp9	1.18	0.879	0.71	0.810	2.33	0.355
Pdgfa	1.27	0.785	1.17	0.587	4.35	0.065
Plat	1.31	0.603	1.41	0.416	0.93	0.814
**Plaur**	**3.59**	**0.002**	2.72	0.342	**20.26**	**0.041**
Plau	2.41	0.127	1.39	0.464	3.94	0.122
Plg	N/A	N/A	N/A	N/A	N/A	N/A
Pten	3.79	0.203	0.605	0.159	4.59	4.59
Ptgs2	1.84	0.79	0.435	0.318	8.44	0.167
Rac1	1.42	0.860	0.94	0.693	0.65	0.752
Rhoa	3.54	0.101	2.97	0.376	**7.69**	**0.043**
Serpine1	5.63	0.111	2.42	0.309	5.00	0.062
Stat3	3.8	0.065	2.19	0.377	4.65	0.172
Tagln	1.32	0.156	0.56	0.801	0.78	0.908
Tgfa	N/A	N/A	N/A	N/A	N/A	N/A
**Tgfb1**	**2.38**	**0.040**	1.6	0.332	2.33	0.361
**Tgfbr3**	**8.11**	**0.006**	3.93	0.222	**6.46**	**0.041**
Timp1	0.29	0.194	0.22	0.168	2.25	0.331
**Tnf**	**3.92**	**0.024**	1.1	0.683	**12.16**	**0.049**
Vegfa	6.54	0.129	3.02	0.369	3.4	0.274
Vtn	1.22	0.721	0.75	0.985	1.23	0.780
Wisp1	1.26	0.693	0.70	0.890	1.23	0.781
Wnt5a	1.89	0.212	1.32	0.493	1.08	0.895

Supplemental Methods

Generation of phage clones

The following oligonucleotide primers (TAG Copenhagen, Copenhagen, Denmark), expressing the indicated peptides, were used to prepare phage clones: tCRK (CRKDK):5´- AAT TCT TGC CGC AAA GAT AAA TAA GGA -3´and 5´- AGC TTC CTT ATT TAT CTT TGC GGC AAG-3´, CRK (CRKDKC): 5´- AAT TCC TGC CGG AAG GAT AAG TGC TA -3´and 5´- AGC TTA CGA CTT ATC CTT CCG GCA GG-3´, CAR (CARSKNKDC): 5´- AAT TCC TGC GCA CGT TCG AAG AAC AAA GAT TGC TA -3´and 5´- AGC TTA GCA ATC TTT GTT CTT CGA ACG TGC GCA GG-3´, where the 5´- end is phosphorylated. The random primer sequences for the negative control phage were 5´- AAT TCC TGC CTT TTG GGG AAG AAT TCG TGC TA -3´and 5´- AGC TTA GCA CGA ATT CTT CCC CAA AAG GCA GG-3´. The oligonucleotide mixture was heated to 95 °C, allowed to cool down to 20 °C in 75 minutes and then cloned into the T7Select 415-1b vector according to the manufacturer's instructions (Merck Millipore, Madison, WI). Individual phage plaques were isolated from the bacterial culture plate and the insert coding region of each recombinant phage was amplified by PCR and checked by DNA sequencing to confirm the clones used for the homing studies as previously described.[2]

Peptide synthesis

Peptides were synthesized with an automated peptide synthesizer by using standard solid-phase fluorenylmethoxycarbonyl chemistry. During synthesis, the peptides were labeled with fluorescein amitide (FAM) using an amino-hexanoic acid spacer as described previously.[3]

Preparation and characterization of iron oxide nanoworms (IONWs)

The iron oxide nanoworms (IONWs) were prepared based on a previously published protocol.[4] Briefly, 0.63 g of $FeCl_3$•$6H_2O$ (Sigma-Aldrich #44944) and 0.25 g of $FeCl_2$•$4H_2O$ (Sigma-Aldrich #44939) were mixed with 4.5 g of Dextran T20 (Pharmacosmos) in 30 ml of deionized water (Millipore). The reaction mixture was cooled to 0 °C. Under a steady flow of nitrogen and vigorous stirring, 1 ml of 28% aqueous ammonium hydroxide (Sigma-Aldrich #338818) was added over 45 minutes. Next, the reaction mixture was heated at 80 °C for 1 h, and cooled to room temperature (RT). After diluting with 90 ml of deionized water, the mixture was centrifuged in 50 ml Falcon tubes at 335 G for 20 min (RT) to remove larger aggregates. The colloidal suspension was transferred to 100,000 MWCO centrifugal filters (Millipore), and centrifuged at 760 G for 30 min (4 °C) – this washing step was repeated 4 times. The dextran was crosslinked with epichlorohydrin (Sigma-Aldrich #540080) in strongly basic conditions (addition of 5 M aqueous NaOH solution). After removing excess epicholohydrin and NaOH with 100,000 MWCO centrifugal filters, the IONWs were aminated with 28% aqueous ammonium hydroxide, dialysed for 48 h, and stored at 4 °C.

FAM coupled tCRK (CRKDK) or scrambled CendR peptide PRP (RPAAPRP) or FAM alone were coupled to the IONWs through a maleimide-PEG(5000)-NHS linker (Jenkem). IONWs were incubated with the linker for 1 h at RT with stirring, washed with PBS in aforementioned centrifugal filters, and then the peptide with free cysteine was added (in excess). After an overnight incubation at RT and final washes, the IONWs were filtered through a 0.22 µm filter, and used within the next week.

Nanoparticle (IONW) targeting study

The nanoparticles (IONWs) coated with either FAM-coupled tCRK, FAM-coupled PRP or FAM alone were dissolved in DPBS. Mice with either 8 or 9 days old excisional wounds or 10 or 11 days old excisional splint wounds were injected with IONW-tCRK (12 mg/kg), IONW-PRP (16 mg/kg) or IONW-FAM (13 mg/kg) intravenously through the tail vein. Five hours after the injection, the mice were perfused with phosphate buffered saline (PBS) containing 1 % bovine serum albumin (BSA) while under deep anesthesia (ketamine-medetomidine according to weight). Skin and skin wounds were excised, embedded in Tissue-Tek OCT medium (Sakura Europe, Alphen aan den Rijn, The Netherlands) and snap-frozen in liquid nitrogen. The samples were then cut and fixed with acetone for immunohistochemistry analysis (IHC). To determine the localization of the IONWs, the sections were double-immunostained with rabbit anti-fluorescein isothiocyanate (FITC) antibody (#71-1900, Invitrogen, Carlsbad, CA) followed by anti-rabbit secondary Alexa Fluor 594 (A11007, Invitrogen, Carlsbad, CA) and with rat anti-mouse CD31 antibody (BD550274, BD Biosciences, San Jose, CA) followed by anti-rat secondary Alexa Fluor 488 (A11070, Life Technologies, Carlsbad, CA). Images were acquired with Zeiss LSM 780 Laser Scanning Confocal Microscope using the same settings throughout the experiments.

Cloning of decorin fusion proteins

Human decorin cDNA[5] without the native signal and pro-peptide sequence were cloned into the mammalian expression vector pEFIRES-P.[6] The tCRK wound homing peptide cDNA was cloned to the C-terminus of decorin flanked by a stop-codon. A 6XHis-tag was cloned to the N-terminus ahead of decorin. The construct was assembled by using the PIPE method[7]. For transformation NEB 5-alpha competent *E. coli* (high efficiency) cells were used (C2987H; New England Biolabs Ipswich, MA) according to the manufacturer's instructions. For plasmid purification (Mini-Prep), PCR purification and agarose gel purification, kits from Qiagen (Hilden, Germany) were used. DCN naturally forms a dimer [8]. The protein sequence of a monomeric 6XHistag-DCN-tCRK fusion protein is: G H H H H H H D E A S G I G P E V P D D R D F E P S L G P V C P F R C Q C H L R V V Q C S D L G L D K V P K D L P P D T T L L D L Q N N K I T E I K D G D F K N L K N L H A L I L V N N K I S K V S P G A F T P L V K L E R L Y L S K N Q L K E L P E K Met P K T L Q E L R A H E N E I T K V R K V T F N G L N Q Met I V I E L G T N P L K S S G I E N G A F Q G Met K K L S Y I R I A D T N I T S I P Q G L P P S L T E L H L D G N K I S R V D A A S L K G L N N L A K L G L S F N S I S A V D N G S L A N T P H L R E L H L D N N K L T R V P G G L A E H K Y I Q V V Y L H N N N I S V V G S S D F C P P G H N T K K A S Y S G V S L F S N P V Q Y W E I Q P S T F R C V Y V R S A I Q L G N Y K G S E F C R K D K Stop.

A schematic map of the DCN-tCRK fusion protein is shown in Fig. 2.

Recombinant protein purification

Cell culture supernatants were filtered and degassed on ice through a 0.45 µm filter unit (Corning #430514, Corning, NY). The 6XHis-tagged proteins were purified by Ni-NTA- IMAC via a two-step purification protocol using first a HisTrap Excel column followed by a HisTrap HP column on the Äkta Start chromatography system (GE Healthcare, Munich, Germany) according to the manufacturer's instructions in a 4 °C cold cabinet. Buffers were prepared from the His Buffer Kit (GE Healthcare/VWR (11-0034-00). All buffers were filtered and degassed.

The HisTrap Excel column eluate was diluted in 20 mM sodium phosphate buffer (pH 7.4) with 0.5 M NaCl to a final imidazole concentration of 30 mM, and then further purified on a HisTrap HP column, with a 35 mM imidazole wash and a gradient elution up to 300 mM imidazole (Fig. S3 includes an example of such a purification chromatogram). The peak fractions were analyzed on a SDS NuPAGE 4-12 % gradient gel (Life Technologies/Thermo Fisher Scientific, Waltham, MA) and visualized via PageBlue Protein Staining Solution (Thermo Fisher Scientific, Waltham, MA).

Selected peak fractions were pooled and dialyzed against cold TBS buffer (pH 7.6) using 50 kDa MWCO Float-A-Lyzers (Fisher Scientific/Spectrum Labs), before concentration via 10 kDa MWCO VivaSpin 6 tubes (GE Healthcare). Samples were filter sterilized (Ultrafree-MC GV Centrifugal Filter 0.22 µm, Millipore, Burlington, MA) and the protein concentration measured at A280 nm via Nanodrop (Thermo Fisher Scientific, Waltham, MA). All steps were performed at 4 °C or on ice. Sterile Tween-20 was added to a final concentration of 0.05% to prevent aggregation, before freezing aliquots rapidly at -80 °C.

Recombinant protein was verified by SDS Page and Western blotting. BioRad's wet tank Mini-PROTEAN Trans-Blot Cell system was used (according to the manufacturer's instructions). A PVDF membrane was probed with a primary murine antibody against human decorin (MAB143, R&D Systems, Minneapolis, MN) according to the manufacturer's protocol. A secondary horseradish peroxidase-coupled anti-mouse antibody from Cell Signaling Technology was used. Chemiluminescent blot images were captured via ImageQuant LAS 4000 mini (GE Healthcare).

Biophysical protein analysis

The hydrodynamic diameter was measured by Dynamic Light Scattering (DLS) using a Zetasizer Nano ZS instrument (Malvern Instruments Ltd, Worchestershire, UK). The DCN-tCRK protein sample was diluted 1:5 in TBS buffer. Three 10X10 s measurements were performed at 25 °C. Data were analyzed using the Zetasizer software v7.11 (Malvern Instruments Ltd.) via the protein analysis model (non-negative least squares analysis followed by L-cuve) and size distribution by volume.

The unfolding temperature of DCN-tCRK was determined using the VP-Capillary DSC (differential scanning calorimetry) instrument (GE Healthcare, Microcal Inc./Malvern Instruments Ltd.) in TBS buffer (50 mM Tris-Cl, 150 mM NaCl, pH 7.5) with a protein concentration of 0.2 mg/ml. All solutions were degassed. Samples were heated from 20 °C to 130 °C at a scanning rate of 2 °C/min. Feedback mode was set to 'low' and the filter period was 5 s. The melting temperature Tm (transition midpoint) was calculated by a Non-2-state fitting model using Origin 7.0 DSC software suite (Microcal Inc.).

Expressed recombinant DCN-tCRK protein was identified from the monomeric gel band using Eksigent 425 NanoLC coupled with Sciex high speed TripleTOF™ 5600+ mass spectrometer. After isolation of gel band and Coomassie stain removal protein was then subjected to reduction (TCEP, 25 mM), alkylation (iodoacetamide, 0.5 M), and trypsin digestion as described in detail in Vähätupa et. al., 2018.[9] After trypsin digestion peptides were diluted to 14 µl of sample buffer (2 % acetonitrile, 0.1 % formic acid) and 1 µl of sample was injected to the triple TOF mass spectrometry.

In vitro binding analyses

In vitro binding of DCN-tCRK and peptides to NRP-1 was analyzed using ELISA analysis. 96-well, black FLUOTRAC™ 600, high binding plates (Greiner Bio-One, Kremsmünster, Austria) were coated with 100µL/well of 100 µg/ml DCN-tCRK in PBS at 4 °C overnight. 10 µg/well RPARPAR and RPAPRARA peptides were coated in parallel as a positive and negative control, respectively. BSA was used as an immobilization control. The plates were washed 3 times with phosphate buffered saline (PBS) and blocked for 1 h at 37 °C with 300 µl of blocking solution (1XPBS, 1% BSA, 0.1% Tween-20). His-tagged neuropilin-1 b1b2 domain (NRP-1 WT) and triple mutant NS346A-E348A-T349A neuropilin-1 b1b2 domain (NRP-1 mutant) were expressed and purified at the Protein Production and Analysis Facility at the Sanford Burnham Prebys Medical Discovery Institute (La Jolla, CA) as described previously.[10] The recombinant proteins NRP1 WT, NRP1 mutant, and DCN-tCRK were FAM (5-(and-6)-Carboxyfluorescein, #90024, Biotium Inc, CA, USA) labeled by mixing 1:10 ratio of amine-reactive FAM dye (diluted in DMSO final concentration 0.2%) and protein. The mixture reaction was incubated in the dark for 2 hours at RT, followed by ultrafiltration/dialysis with PBS to separate free dye from the protein. 100 µl of FAM-labeled NRP1 WT or NRP1 mutant protein in blocking solution was added to each well (20 µg/well), incubated at room temperature for 4-6 hours at room temperature or 4 °C overnight, and washed 3 times with blocking solution. After adding 100 µl PBS in each well, the plate was immediately read with top read mode using a fluorescence reader (Flex Station II, Molecular Devices; peak excitation = 485 nm, peak emission =530 nm, cut off =515).

For the binding of FAM-DCN-tCRK to NRP-1 positive prostate carcinoma-3 (PC-3) cells (gift from the Ruoslahti laboratory at Sanford-Burnham-Prebys Medical Discovery Institute, La Jolla, CA) and negative melanoma (M21) cells (gift from David Cheresh Lab at University of California San Diego, La Jolla, CA) *in vitro*, the cells were first cultured in growth medium composed of 10% fetal bovine serum (FBS) in DMEM high glucose medium supplemented with penicillin, and streptomycin (Gibco). For experiments, the medium was aspirated, the cells were washed twice with warm medium, and fresh medium was added along with 10 µg FAM-labeled DCN-tCRK recombinant protein. The labelling was done by directly coupling DCN-tCRK recombinant protein to Fluorescein using Lightning-Link Fluorescein kit (Expedon Ltd, UK) according to the manufacturer protocol. The cells were incubated at 37 °C for one hour; medium was aspirated, the cells were washed and fixed with −20 °C methanol. The cells were washed with PBS and blocked (PBS, 1% BSA, 1% FBS, 1% goat serum, 0.05% Tween-20) for 30 minutes at RT followed by primary anti-FITC (Invitrogen, CA, USA. Catalog # A-889) for one hour at RT. The cells were washed, and secondary antibodies Alexa Fluor 488 goat anti-rabbit IgG (Invitrogen, USA) were applied for one hour at RT in the dark. The nuclei of cells were stained with DAPI. The coverslips were mounted on glass slides with Fluoromount-G (Electron Microscopy Sciences, PA, USA), imaged using confocal microscopy (Olympus FV1200MPE, Tokyo, Japan) and analyzed using the FV10-ASW4.2 viewer.

Recombinant protein pharmacokinetics

Recombinant proteins DCN-tCRK or DCN were diluted in Tris buffered saline (TBS) containing 0.05 % Tween-20. The pharmacokinetics of DCN-tCRK and DCN were studied with 8 week old Balb/c male mice. 5 mg/kg either DCN-tCRK or DCN was injected in tail vein under isoflurane anesthesia. Blood samples from distinct tail vein were gathered at 15 min, 30 min, 60 min, 2 h, 4 h, and 16 h after injection. At 8 h or 24 h after the injection, the mice were sacrificed under medetomidine-ketamine anesthesia and blood samples were collected from the subclavian vein. The samples were mixed with 1 M ethylenediaminetetraacetic acid (EDTA), centrifuged 2000 g for 10 min at room temperature and the plasma was stored for analysis. The concentration of human origin decorin in the plasma samples was determined with Human Decorin DuoSet ELISA kit (#DY143, R&D Systems, Minneapolis, MN) according to instructions provided by the manufactured. A venous blood sample from an uninjected mouse was used in each plate to ensure the specificity of the primary antibody.

Collagen lattice contraction assay

Human normal fibroblasts and RDEB patient-derived fibroblasts were cultured in DMEM supplemented with 10% FBS, as previously described.[11] The collagen lattices were prepared by mixing the cell suspension with neutralized rat tail collage type I (Advance BioMatrix, Carlsbad, CA). The final concentration of collagen was 2.4 mg/ml with a cell density of 2.1 x 10^5 cells/ml. 500 µl of cells/collagen suspension was dispensed into a single well of 24-well plate and

allowed to solidify for 30 min at room temperature. 0.5 ml of DMEM supplemented with 5% of FBS was added in each well after collagen polymerization and plates were cultured at 37ºC with 5% CO_2. After 12 hours of incubation, the gel from each well was gently released by the thin pipet tip and DCN or DCN-CRK were added respectively at a final concentration 75 µM (n=3 per condition). Images were acquired at 12 hours (initial area) and 48 hours (contraction area) respectively and the areas of gels were quantitated using Image J.

Supplemental references

1. Y. Liao, L. Ivanova, R. Sivalenka, T. Plumer, H. Zhu, X. Zhang, *et al.* (2018). Efficacy of Human Placental-Derived Stem Cells in Collagen VII Knockout (Recessive Dystrophic Epidermolysis Bullosa) Animal Model. *Stem Cells Transl Med* **7**: 530-542.
2. T. A. Järvinen (2012). Design of target-seeking antifibrotic compounds. *Methods Enzymol* **509**: 243-261.
3. T. Urakami, T. A. Jarvinen, M. Toba, J. Sawada, N. Ambalavanan, D. Mann, *et al.* (2011). Peptide-directed highly selective targeting of pulmonary arterial hypertension. *Am J Pathol* **178**: 2489-2495.
4. J. H. Park, G. von Maltzahn, L. Zhang, M. P. Schwartz, E. Ruoslahti, S. N. Bhatia, *et al.* (2008). Magnetic Iron Oxide Nanoworms for Tumor Targeting and Imaging. *Adv Mater* **20**: 1630-1635.
5. T. Krusius and E. Ruoslahti (1986). Primary structure of an extracellular matrix proteoglycan core protein deduced from cloned cDNA. *Proc Natl Acad Sci U S A* **83**: 7683-7687.
6. S. Hobbs, S. Jitrapakdee and J. C. Wallace (1998). Development of a bicistronic vector driven by the human polypeptide chain elongation factor 1alpha promoter for creation of stable mammalian cell lines that express very high levels of recombinant proteins. *Biochem Biophys Res Commun* **252**: 368-372.
7. H. E. Klock and S. A. Lesley (2009). The Polymerase Incomplete Primer Extension (PIPE) method applied to high-throughput cloning and site-directed mutagenesis. *Methods Mol Biol* **498**: 91-103.
8. P. G. Scott, P. A. McEwan, C. M. Dodd, E. M. Bergmann, P. N. Bishop and J. Bella (2004). Crystal structure of the dimeric protein core of decorin, the archetypal small leucine-rich repeat proteoglycan. *Proc Natl Acad Sci U S A* **101**: 15633-15638.
9. M. Vähätupa, J. Nättinen, A. Jylhä, U. Aapola, M. Kataja, P. Koobi, *et al.* (2018). SWATH-MS Proteomic Analysis of Oxygen-Induced Retinopathy Reveals Novel Potential Therapeutic Targets. *Invest Ophthalmol Vis Sci* **59**: 3294-3306.
10. T. Teesalu, K. N. Sugahara, V. R. Kotamraju and E. Ruoslahti (2009). C-end rule peptides mediate neuropilin-1-dependent cell, vascular, and tissue penetration. *Proc Natl Acad Sci U S A* **106:** 16157-16162
11. Y. Liao, L. Ivanova, H. Zhu, T. Plumer, C. Hamby, B. Mehta, *et al.* (2018). Cord Blood-Derived Stem Cells Suppress Fibrosis and May Prevent Malignant Progression in Recessive Dystrophic Epidermolysis Bullosa. *Stem Cells* **36**: 1839-1850.
